# Latin American databases of natural products: biodiversity and drug discovery against SARS-CoV-2†

**DOI:** 10.1039/d1ra01507a

**Published:** 2021-05-04

**Authors:** Marvin J. Núñez, Bárbara I. Díaz-Eufracio, José L. Medina-Franco, Dionisio A. Olmedo

**Affiliations:** Natural Product Research Laboratory, School of Chemistry and Pharmacy, University of El Salvador San Salvador El Salvador marvin.nunez@ues.edu.sv; DIFACQUIM Research Group, Department of Pharmacy, School of Chemistry, National Autonomous University of Mexico Mexico City 04510 Mexico jose.medina.franco@gmail.com dieb@comunidad.unam.mx; Center for Pharmacognostic Research on Panamanian Flora (CIFLORPAN), College of Pharmacy, University de Panama Panama ciflorp4@up.ac.pa; Sistema Nacional de Investigación (SNI), SENACYT Panamá

## Abstract

In this study, we evaluated 3444 Latin American natural products using cheminformatic tools. We also characterized 196 compounds for the first time from the flora of El Salvador that were compared with the databases of secondary metabolites from Brazil, Mexico, and Panama, and 42 969 compounds (natural, semi-synthetic, synthetic) from different regions of the world. The overall analysis was performed using drug-likeness properties, molecular fingerprints of different designs, two parameters similarity, molecular scaffolds, and molecular complexity metrics. It was found that, in general, Salvadoran natural products have a large diversity based on fingerprints. Simultaneously, those belonging to Mexico and Panama present the greatest diversity of scaffolds compared to the other databases. This study provided evidence of the high structural complexity that Latin America's natural products have as a benchmark. The COVID-19 pandemic has had a negative effect on a global level. Thus, in the search for substances that may influence the coronavirus life cycle, the secondary metabolites from El Salvador and Panama were evaluated by docking against the endoribonuclease NSP-15, an enzyme involved in the SARS CoV-2 viral replication. We propose in this study three natural products as potential inhibitors of NSP-15.

## Abstract 2

We performed a cheminfomatic analysis of four databases in Latin America and evaluated its potential *in silico* against SARS-CoV-2.

## Graphical abstract

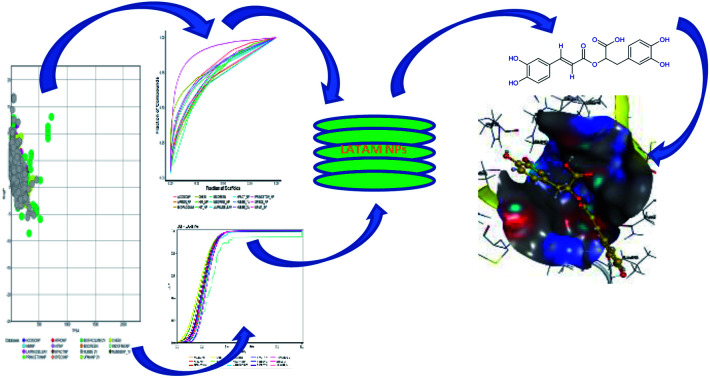

## Introduction

Natural products (NPs) and their derivatives continue to be an important source of chemical entities for the design and development of drugs, reporting a total of 355 molecules approved for clinical use between the years 1981 to 2019 and placing them at an intermediate point between biological products (262).^1^ Databases of publicly available NPs have been used in process discoveries, and to develop drug design and contain metabolites of plants, fungi, marine organisms, and bacteria.^2^ These databases contain a variety of chemical types that have various pharmacological activities.^3^ Some natural products from these databases have been evaluated by chemo- and bioinformatic methods, concluding that they have large structural and scaffold diversities.^4^

Latin American database of natural products (LATAM_DBS_NPs) has been evaluated using chemoinformatics and bioinformatics tools. Such analysis allowed us to create and manipulate molecules by representing and visualizing the chemical structures of small molecules. Furthermore, we calculate physicochemical properties based on chemical descriptors, chemical space, fingerprints, similarity studies, the content of cyclic systems, fragments of aliphatic and alicyclic skeletons applied to the bases of natural products.^5–14^ The increasing use of cheminformatics in NPs research has led to the sub-discipline “natural product informatics.”^15^ In this study we analyzed and compared four databases of Latin American NPs: LAIPNUDLSAV, UPMA_2V, BIOFACQUIM_2V, NUBBE_2V and eleven databases of natural products available for free at http://zinc12.docking.org/browse/catalogs/. These include the following datasets: AfroDb NPs; Herbal ingredients *in vivo* metabolism database (HIM); Herbal Ingredients' Targets (HIT); ICC Indofine NPs DBs (Indofine Chemical Company); Naturally occurring Plant based Anti-cancerous Compound-Activity-Target Database (NPACT Database); AnalytiCon Discovery NP (ACDISC_NP); Specs Natural Products; The Nuclei of Bioassays, Ecophysiology and Biosynthesis of Natural Products Database (NuBBE DB 2013 and 2017); InterBioScreen Ltd (IBScreen NP); Chemical Entities of Biological Interest (ChEBI); and Princeton BioMolecular Research.

This is the first systematic cheminformatic and bioinformatic study of LATAM_DBS_NPs against NPS-15 endoribonuclease of SARS CoV-2. The acronym refers to severe acute respiratory syndrome (SARS) and the CoV-2 refers to type 2 coronavirus.

NPs have been found to be highly potent in blocking enzyme function and membrane receptors of human coronavirus.^16^ In addition, the evolution of COVID-19 is featured with uncontrolled inflammation, threeway anti-inflammatory herbal compounds will be a potential tool to repress such fatal symptoms.^17^ Nsp-15, a hexamer endoribonuclease that cleaves the remains of uridines. This protein is homologous to the Nsp15 endoribonucleases of SARS-CoV and MERS-CoV but differs from them in that it can contribute to the increased virulence of SARS-CoV-2.^18^

## Materials and methods

### Databases of natural products

LATAM_DBS_NPs and REF_NPs were analyzed for their physicochemical properties, chemical space, scaffold content, and molecular complexity. These include two datasets prepared in house and another two NPs DBs published in journals: LAIPNUDELSAL, UMPA_NP_2V, BIOFACQUIM_2V, and NUBBE_2V DB, that were compared with eleven NPs of the reference data set (REF_NPs) of public access: AFRON_NP, AnalytiCon DB ACDISC_NP, CHEBI, HITM_NP, HIT_NP, IBSCREEN_NP, INDOFINE_NP, NPACT_NP, PRINCETON_NP, SPECS_NP and NUBBEDB V1 and V2. Table 1 indicates the compounds analyzed and Table 2 shows the description and sources of the databases used in this work.

**Table 1:** Natural products databases analysed in this work

Dataset of NPs	Number of initial compounds	Number of compounds after data curation
LAIPNUDELSAL_NP	214	196
UPMA_2V_NP	485	462
BIOFACQUIM_NP	531	507
NUBBE_2V_NP	2366	2279
IBSCREEN_NP subset	8444	8413
CHEBI subset	6054	5622
AFRO_NP	550	494
ACDISC_NP	11 245	10 859
HIM_NP	663	637
HIT_NP	801	583
INDOFINE_NP	143	99
NPACT_NP	1422	1093
SPECS_NP subset	1488	1349
PRINCETON subset	14 083	13 672
NUBBE_1V	587	148
	49 076	46 413

**Table 2:** Description and sources of natural products DBs used in this research

Dataset of NPs	Description	Sources
LAIPNUDELSAL_NP	This database was built by making a bibliographic compilation of the compounds isolated and characterized by spectroscopic methods of the flora of El Salvador during the period 1981 to 2019	In house (Laboratorio de Investigación en Productos Naturales de la Universidad de El Salvador, El Salvador)
UPMA_2V_NP	A collection actualized of NPs isolated from Panamanian flora from 1972 to 2019	In house (CIFLORPAN, Facultad de Farmacia, Universidad de Panamá, Panamá)
BIOFACQUIM_NP_2V	Database with NPs isolated and characterized in Mexico from 2020	DOI: 10.3390/biom9010031^7^
NUBBE_2V_NP	NuBBE_DB_2V has compounds obtained from plants, semi-synthetic products microorganisms, products of biotransformation, and marine environment modified in 2017	DOI: 10.1038/s41598-017-07451-x,^19^ available in: https://nubbe.iq.unesp.br/portal/nubbe-search.html
IBSCREEN_NP subset	Natural products and derivatives NPs were generated for institutes of the Soviet Union from 2015	Available in: http://zinc12.docking.org/browse/catalogs/natural-products, h commercially available: https://www.ibscreen.com/natural-compounds^20^
CHEBI subset	Chemical entities of biological interest (ChEBI). This dictionary includes natural products, synthetic compounds, drugs, and chemicals environment	DOI: 10.1093/nar/gkp886,^21^ commercially available in: https://www.ebi.ac.uk/chebi/
AFRO_NP	AfroDb represents a collection of NPs from the African continent from 2013	DOI: 10.1371/journal.pone.0078085,^22^ available in: http://zinc12.docking.org/browse/catalogs/natural-products
ACDISC_NP	Natural products bioactive from 2013, AnalytiCon Discovery NP	Available in: http://zinc12.docking.org/browse/catalogs/natural-products,^23^http://zinc.docking.org/catalogs/acdiscnp/
HIM_NP	HIM Db is set up for the *in vivo* metabolite's information of the active ingredients for Chinese herbs from 2013	DOI: 10.1186/1758-2946-5-28,^24^ available in: http://zinc12.docking.org/browse/catalogs/natural-products
HIT_NP	HIT is collecting target information of herbal active compounds from 2013	DOI: 10.1093/nar/gkq1165,^25^ available in: http://zinc12.docking.org/browse/catalogs/natural-products
INDOFINE_NP	Databases of NPs from 2013	Available in: http://zinc12.docking.org/browse/catalogs/natural-products,^26^http://zinc12.docking.org/catalogs/indofinenp
NPACT_NP	Database of plant-derived natural compounds that exhibit anti-cancerous activity from 2013	DOI: org/10.1093/nar/gks1047,^27^http://zinc12.docking.org/catalogs/npactnp
SPECS_NP	A collection of NPs from 2015	Available in: http://zinc12.docking.org/catalogs/specsnp,^28^ commercially available in http://www.specs.net
PRINCETON subset	Compounds obtained for high throughput screening	Available in: http://zinc12.docking.org/browse/catalogs/natural-products,^29^ commercially available in: http://www.princetonbio.com
NUBBE_1V	The Nuclei of Bioassays, Ecophysiology, and Biosynthesis of Natural Products Database (NuBBEDB_1V) was created as the first natural product library from Brazilian biodiversity in 2013	DOI: 10.1021/np3006875,^30^ available in: http://zinc12.docking.org/catalogs/nubbenp

The molecular descriptors were determined by MOE v.2018.0102 (ref. 31) and DataWarrior v.5.2.1:^32^ hydrogen bond acceptors (HBAs), hydrogen bond donors (HBDs), number of rotatable bonds (NRBs), the octanol/water partition coefficient (w/o; *S* log *P*), molecular weight (MW), and topological polar surface area (TPSA) (data in ESI†). These descriptors have been widely used to describe properties of interest in research and development departments in the pharmaceutical industries.^5–14^

### Chemical space

A visual representation of the chemical space was done using the principal component analysis (PCA)^5–14^ of the six physicochemical properties with DataWarrior v.5.2.1.^32^

### Molecular fingerprints

The molecular fingerprints have been used as indicators of structural diversity in different data sets.^33–35^ Two fingerprint keys were calculated: Extended Connectivity Fingerprints of Radius six (ECFP-6) and Molecular Access System (MACCS) Key (166-bits) with the Python script.^36^ The correlation of similarity pairs was calculated with the correlation-like-similarity index: Tanimoto coefficient and cosine-like similarity index were analyzed with a cumulative distribution function (CDF).

### Molecular scaffolds diversity

The Bemis–Murcko scaffold references were calculated with molecular equivalent indices (MEQI)^37–39^ and DataWarrior.^31^ MEQI was used to obtain the cyclic system ring of the compounds in the different databases of NPs for their scaffold content and diversity.^40–43^

The scaffold distribution of the NPs dataset was explored, evaluated, and plotted in RStudio^44,45^ for the cyclic system retrieval curves (CSR) for indicated the scaffold diversity of different databases.^7–14^ These curves were plotted with the fraction of scaffolds in the *x*-axis and the fraction of compounds in the *y*-axis. The CSR curves provide information on the scaffold diversity of all the data sets.

### Molecular complexity

The molecular complexity of NP datasets was explored and calculated using the descriptors: a fraction of sp3 hybridized carbons (Fsp3), fraction of chiral carbon (FCC), fraction of atoms aromatic (Fa_Aro), fraction of aromatic bond (Far_b), molecular flexibility, shape index and globularity molecular.^11,14,46,47^

### Molecular docking simulation

#### Preparation of the structure of endoribonuclease NSP-15

The endonucleases NSP-15 (PDB ID: 6WXC) were obtained from the Protein Data Bank.^48^ The protein preparation procedure consisted of eliminating the water molecules, then we checked and corrected possible errors in the primary amino acid sequences using the sequence editor window in the MOE module. Then, using the Quickpre option, hydrogens were added, the protein–ligand complex was corrected, protons were added to the ligand and the protein in 3D model; we selected the MMFF94x force field, then the partial charges were established, and the minimum conformation was generated. This protein was optimized; it was saved in dock_moe format.

The binding site was checked with the option (Site Finder), which is listed by the size of the possible binding sites of the ligand to the protein, in the first four rows appear the amino acid residues that maintain the interaction with the co-crystallized ligand. This step is important since other sites where there is no compound can be selected to simulate docking molecular. Finally, we isolated the atoms and the region of proximity to the selected site in the protein, reviewed the structure of the ligand in CPK format, and generated the surface area of the binding site.^49^

#### Preparation of natural product databases

The preparation of NPs was carried out with the module “preparing of small molecule dataset”.

The database was created in the excel format and later transformed into a CVS file. This was imported into the MOE window, where we checked if the structures were correctly drawn in their 2D model. Then, we saved it in a format with moe extension. This constituted the working database for chemoinformatic analysis mbd format.

For curing of the databases, we removed the compound duplicates, washed for removing protonated forms of acidic and basic, disconnected the metals present, reviewed the presence of partial loads, and finished minimizing energy state structures and generated conformations 3D, for subsequent analyzes.^49^

#### Protocol of docking molecular

The molecular docking simulation protocol was performed using the following procedure: first, we opened the protein that we have in moe format and after the dock panel in windows MOE. Herein, we selected the MOE option with de parameter: receptor, atoms receptor. In the box site, we chose atoms ligand and the databases of NPs in format mdb.

In the tab that indicates the method and scoring function, we chose put in the placement method: Triangle Matcher con the score London dG and number pose in thirty, while that in refinement, we selected a rigid receptor and the scoring GBVI/WSA with five poses. The forcefield used were MMFF94x.^49,50^

## Results and discussion

### Chemical space

**Fig. 1:**
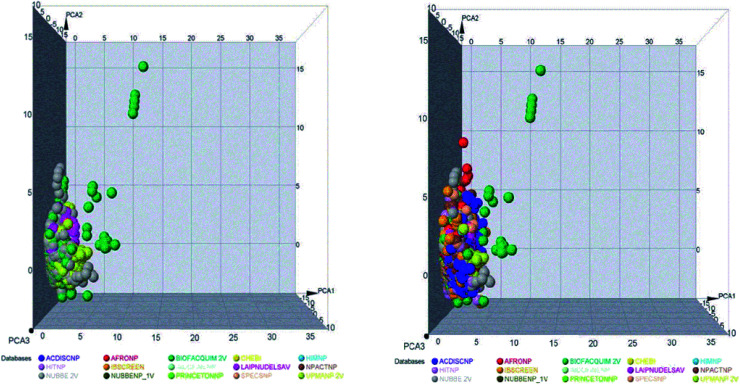
Representation of the chemical space of four LATAM_DBS_NPs (left) and LATAM_DBS_NPs with the eleven REF_NPs (right).

**Fig. 2:**
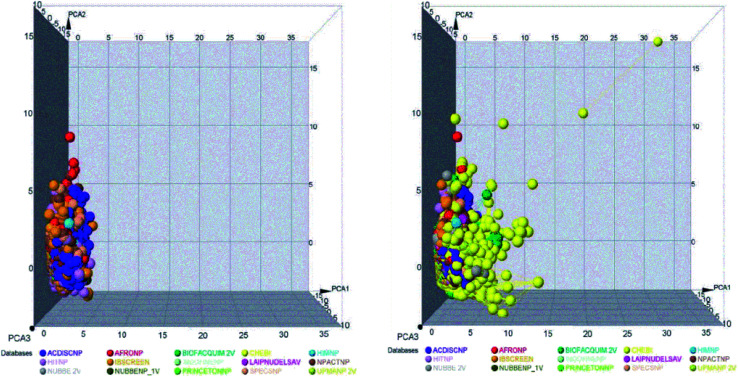
Representation of the chemical space of all NPs, REF_NPs (left) and overlap of all databases evaluated (right).

The space chemicals in 46 413 unique compounds were visualized using PCA of descriptors physiochemical properties. The visualization of space chemicals shows that LATAM_DBS_NPs, and REF_NPs occupy similar chemical spaces. Fig. 1 and 2 show a visual representation of the chemical space of 3440 compounds of LATAM_DBS_NPs. The difference in the variance of the six physicochemical properties analyzed by PCA, is present in ESI.†

These data indicate that the partition coefficient (*S* log *P*) and TPSA have the greatest effect on the variance statistic with a value of 66.69% and 22.39%, respectively, while others descriptor of physicochemical properties has a much lower influence on the envelope, showing a value of 6.27%.

### Fingerprint-based diversity

The molecular diversity of LATAM_DBS_NP and REF_NPs were calculated using the MACCS and ECFP-6 fingerprint (FP) and the Tanimoto and Cosine similarity index.

The graphic of diversity analysis on the *x*-axis is the similarity value pairs based on ECFP-6/Tanimoto, ECFP-6/Cosine, MACCS/Tanimoto/, MACCS/Cosine index, while on the axis of the ordinate we plotted the CDF values for each database evaluated. The CDF realized with Tanimoto/ECFP-6 indicated that LAIPNUDELSAV and PRINCETON_NP datasets are the most diverse, while that with Cosine/ECFP-6 the INDOFINE_NP, LAIPNUDELSAV, PRINCETON_NP and UPMA_2V databases demonstrated better diversity based on fingerprint (ESI†).

The analysis of MACCS fingerprint and Tanimoto similarity indicated that LAIPNUDELSAV, PRINCETON_NP and INDOFINE_NP are more diverse and HIT NP and CHEBI are the least diverse; meanwhile, the metric MACSS/Cosine similarity index, HIMNP indicated greater diversity as the database reference while LATAM_DBS_NPs and REF_NP showed median values greater than 0.50, except for NUBBE_2V, SPECSNP and UPMANP2V which were less diverse, according to MACCS keys/Cosine similarity index. Tables 3 and 4 show summary statistics of the pairwise similarity computed with MACCS/Tanimoto and MACCS/Cosine.

**Table 3:** Summary statistics of the pairwise similarity computed with MACCS/Tanimoto^a^

NPS_DBS	Mean	SD	Min	1st Q	Median	3rd Q	Max
ACDISC NP	0.4883	0.1426	0.0000	0.3860	0.4821	0.5849	1.0
AFRO NP	0.4634	0.1709	0.0000	0.3462	0.4694	0.5818	1.0
BIOFACQUIM	0.4534	0.1447	0.0172	0.3529	0.4444	0.5429	1.0
CHEBI	0.2935	0.1473	0.0	0.1875	0.2821	0.3857	1.0
HIM NP	0.4547	0.1647	0.0294	0.3333	0.4340	0.5614	1.0
HIT NP	0.3836	0.1845	0.00	0.2537	0.375	0.5000	1.0
IBSCREEN	0.4614	0.1309	0.0000	0.3704	0.4627	0.5507	1.0
INDOFINENP	0.5022	0.1973	0.0870	0.3684	0.4681	0.5778	1.0
LAIPNUDELSAV	0.5978	0.1539	0.1277	0.4902	0.5870	0.7045	1.0
NPACTNP	0.4842	0.1633	0.0000	0.3778	0.4884	0.5962	1.0
NUBBE 1V	0.4362	0.1653	0.0000	0.3214	0.4286	0.5385	1.0
NUBBE 2V	0.4243	0.1728	0.0000	0.3061	0.4259	0.5410	1.0
PRINCETONNP	0.5176	0.1329	0.0429	0.4237	0.5000	0.6000	1.0
SPECSNP	0.4790	0.1598	0.0400	0.3594	0.4638	0.5926	1.0
UPMANP 2V	0.4885	0.1608	0.0000	0.3768	0.4744	0.5926	1.0

a Min: minimum; Max: maximum; Q: quartile; SD, standard deviation.

**Table 4:** Summary statistics of the pairwise similarity computed with MACCS/Cosine^a^

NPS_DBS	Mean	SD	Min	1st Q	Median	3rd Q	Max
ACDISC NP	0.5674	0.2310	0.0211	0.3747	0.5628	0.7968	1.0000
AFRO NP	0.5590	0.2287	0.0294	0.3711	0.5483	0.7757	1.0000
BIOFACQUIM	0.5231	0.2224	0.0416	0.3425	0.5038	0.7280	1.0000
CHEBI	0.4783	0.2070	0.0047	0.3214	0.4616	0.6259	1.0000
HIM NP	0.6139	0.2166	0.0197	0.4299	0.6406	0.8071	1.0000
HIT NP	0.5573	0.2238	0.0178	0.3730	0.544	0.7177	1.000
IBSCREEN	0.5666	0.2576	0.1987	0.3136	0.5502	0.8004	0.9083
INDOFINE NP	0.5059	0.2558	0.1004	0.2961	0.5511	0.6273	1.0000
LAIPNUDELSAV	0.5743	0.2645	0.1916	0.3705	0.5423	0.8362	0.9785
NPACT NP	0.5298	0.1606	0.2839	0.4056	0.5639	0.6240	0.8189
NUBBE 1V	0.5261	0.1577	0.2557	0.3869	0.5219	0.6464	0.8839
NUBBE 2V	0.4475	0.2088	0.0972	0.3094	0.4252	0.5601	0.8756
PRINCETONNP	0.5411	0.1530	0.2328	0.4680	0.5262	0.6440	0.8626
SPECSNP	0.4191	0.2066	0.1567	0.2651	0.3416	0.5419	0.9026
UPMANP 2V	0.5212	0.1842	0.1731	0.3789	0.4936	0.6729	0.9360

a Min: inimum; Max: maximum; Q: quartile; SD, standard.

### Scaffold diversity

**Fig. 3:**
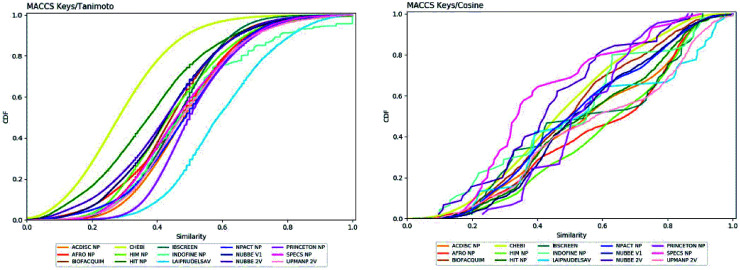
Cumulative distribution function of the pairwise similarity distribution of the different data sets computed with MACCS-Tanimoto and Cosine.

The diversity of scaffolds of Latin American NPs was based on the Murcko scaffold and Murcko skeleton was obtained with RStudio.^45,46^ The Murcko scaffold contains all plain ring systems of the given molecules, plus all direct connections between them. Substituents, which do not contain ring systems are removed from rings and ring connecting chains, while that in the Murcko skeleton was a generalized Murcko scaffold, which has all heteroatoms replaced with carbon atoms (Fig. 3).^31^

Table 5 summarizes the results of the scaffold diversity of the fifteen databases. In this table, the number and fraction of scaffolds are reported, the number and fraction of scaffolds containing only one compound (singletons), and metrics were obtained from CSR curves (AUC and *F*_50_). According to these metrics, databases: NPACT_NP, AFRODB, BIOFACQUIM_2V, and UPMA_2V are those having the largest fractions of scaffolds (*FN*/*M*) with values equal to or greater than 0.46.

**Table 5:** Summary of the scaffold diversity (ring System of the compound data sets)^a^

Databases	*M*	*N*	*FN*/*M*	*N*_sing_	*FN*_sing_/*M*	*FN*_sing_/*N*	AUC	*F*_50_
LAIPNUDELSAV	196	76	0.3878	40	0.2041	0.5263	0.7301	0.1579
UPMA_2V	462	215	0.4654	129	0.2792	0.6000	0.7096	0.1907
BIOFACQUIM_2V	507	239	0.4714	144	0.2840	0.6025	0.7739	0.1042
NUBBE_2V	2279	720	0.3159	349	0.1531	0.4847	0.7633	0.1177
IBSCREEN	8413	2881	0.3424	1449	0.1722	0.5030	0.7890	0.0415
CHEBI	5622	2171	0.3862	1637	0.2912	0.7540	0.7096	0.1621
AFRONP	494	253	0.5121	180	0.3644	0.7115	0.7988	0.0950
ACDISCNP	10 859	1663	0.1531	405	0.0373	0.2435	0.7208	0.2059
HIM_NP	637	136	0.2135	30	0.0471	0.2206	0.7287	0.1959
HIT_NP	583	194	0.3328	77	0.1321	0.3969	0.7115	0.2000
INDOFINE_NP	99	30	0.3030	13	0.1313	0.4333	0.7052	0.1702
NPACT_NP	1093	564	0.5160	390	0.3568	0.6915	0.7499	0.1593
SPECS_NP	1349	364	0.2698	133	0.0986	0.3654	0.9046	0.0255
PRINCETON_NP	13 672	1020	0.0746	396	0.0290	0.3882	0.7736	0.0962
NUBBE_1V	148	52	0.3514	31	0.2095	0.5962	0.7100	0.1841

a *N*: number of chemotypes; *M*: number of molecules; *N*_sing_: number of singletons; AUC: area under the curve; *F*_50_: fraction of chemotypes that contains 50% of the data set.

**Fig. 4:**
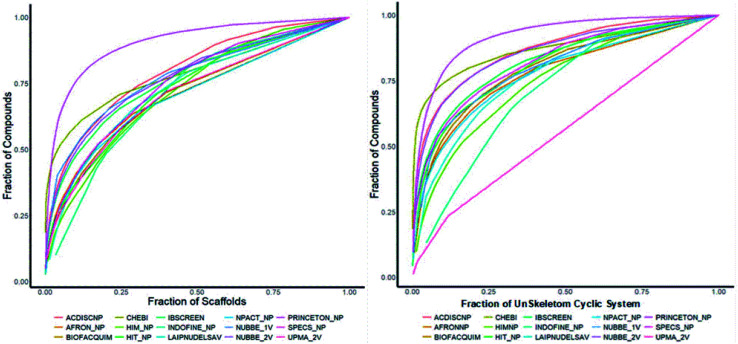
Murcko scaffold (a) and Murcko skeleton (b) retrieval curves for the data sets studied in this work.

Fig. 4(a) shows the metric of Murcko scaffold (ring system) in the fifteen databases that correlated with CSR curves, *F*_50_ (a fraction of chemotypes that contains 50% of the data set), and areas under the curve (AUC) are reported in Table 5. These curves indicate that the Latin American dataset UPMA_2V and NUBBE_1V are those with the greatest structural diversities with *F*_50_ 0.19 and 0.18, respectively. On the contrary, when comparing the values of AUC 0.90, 0.79. 0.78 and 0.77, we observe that the databases SPECS_NP, AFRODB, IBSCREEN, BIOFACQUIM_2V and PRINCETON_NP are the least diverse.

However, Fig. 4(b) shows the CRS curves of skeleton cyclic system and AUC and *F*_50_ metrics. The CSR curves indicate that SPECS_NP, AFRONNP, IBSCREEN, AFRONNP, BIOFACQUIM_2V and NUBBE_2V are the least diverse when comparing their values of AUC 0.92, 0.87, 0.86, 0.85 and 0.82, respectively, while when comparing the values of *F*_50_, it was observed that the database UPMA_2V (0.42) and HIT_NP are the most diverse in its content from the Murcko skeleton, and ACDISCNP, LAIPNUDELSAV and INDOFINE_NP have diversity Murcko skeleton intermediate values from 0.10 to 0.15.

Meanwhile, Table 6 summarizes the diversity of the Murcko skeleton (unskeleton cyclic system) of the fifteen databases. This table presents the number and fraction of unskeleton cyclic systems (Mucko skeleton), the number and fraction of containing only one compound (singletons), and metrics obtained from CSR curves (AUC: area under the curve; *F*_50_: fraction of chemotypes that contains 50% of the data set.)

**Table 6:** Summary of the Murcko skeleton diversity (unskeleton cyclic system of the compound data sets)^a^

Databases	*M*	*N*	*FN*/*M*	*N*_sing_	*FN*_sing_/*M*	*FN*_sing_/*N*	AUC	*F*_50_
LAIPNUDELSAV	196	46	0.2347	17	0.0867	0.3696	0.7717	0.1304
UPMA_2V	462	401	0.8680	355	0.7684	0.8853	0.5601	0.4239
BIOFACQUIM_2V	507	134	0.2643	66	0.1302	0.4925	0.8574	0.0395
NUBBE_2V	2279	354	0.1553	146	0.0641	0.4124	0.8240	0.0624
IBSCREEN	8413	1668	0.1983	645	0.0767	0.3867	0.8723	0.0075
CHEBI	5622	1206	0.2145	830	0.1476	0.6882	0.7777	0.0964
AFRONNP	494	166	0.3360	99	0.2004	0.5964	0.8665	0.0334
ACDISCNP	10 859	898	0.0827	186	0.0171	0.2071	0.7512	0.1566
HIMNP	637	83	0.1303	8	0.0126	0.0964	0.8081	0.0796
HIT_NP	583	113	0.1938	42	0.0720	0.3717	0.7079	0.2273
INDOFINE_NP	99	22	0.2222	7	0.0707	0.3182	0.7767	0.1034
NPACT_NP	1093	348	0.3184	184	0.1683	0.5287	0.8217	0.0698
SPECS_NP	1349	215	0.1594	70	0.0519	0.3256	0.9202	0.0247
PRINCETON_NP	13 672	486	0.0355	145	0.0106	0.2984	0.7945	0.0833
NUBBE_1V	148	36	0.2432	14	0.0946	0.3889	0.7956	0.0896

a *N*: number of chemotypes; *M*: number of molecules; *N*_sing_: number of singletons; AUC: area under the curve; *F*_50_: fraction of chemotypes that contains 50% of the data set.

### Scaffold content in Latin American databases of natural product

**Fig. 5:**
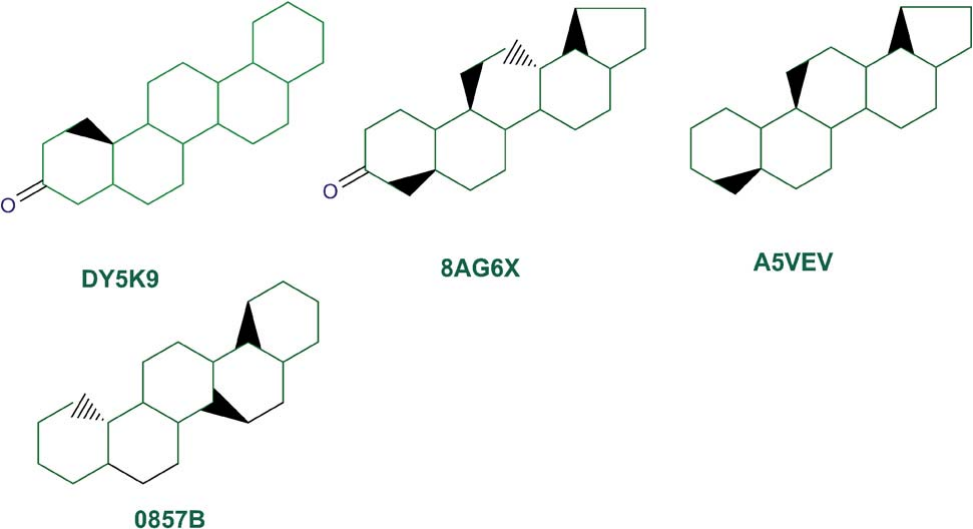
Murcko scaffolds identified in LAIPNUDELSAV, BIOFACQUIM and UPMA_2V.

**Fig. 6:**
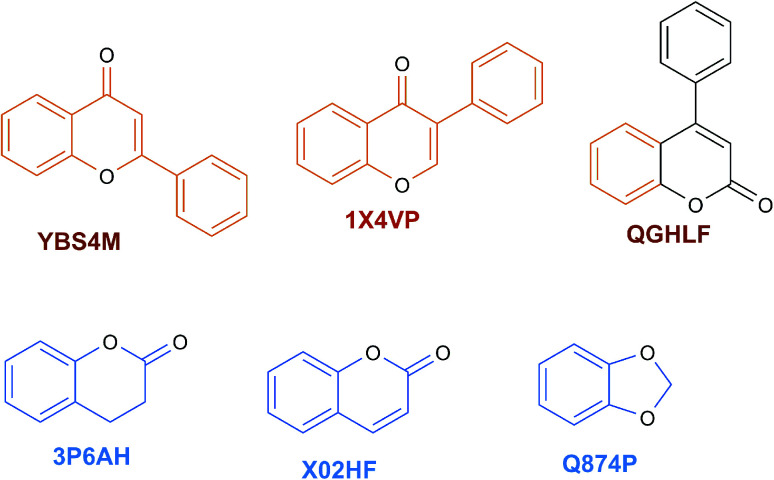
Scaffolds identified in LATAM_DBS_NPs.

**Fig. 7:**
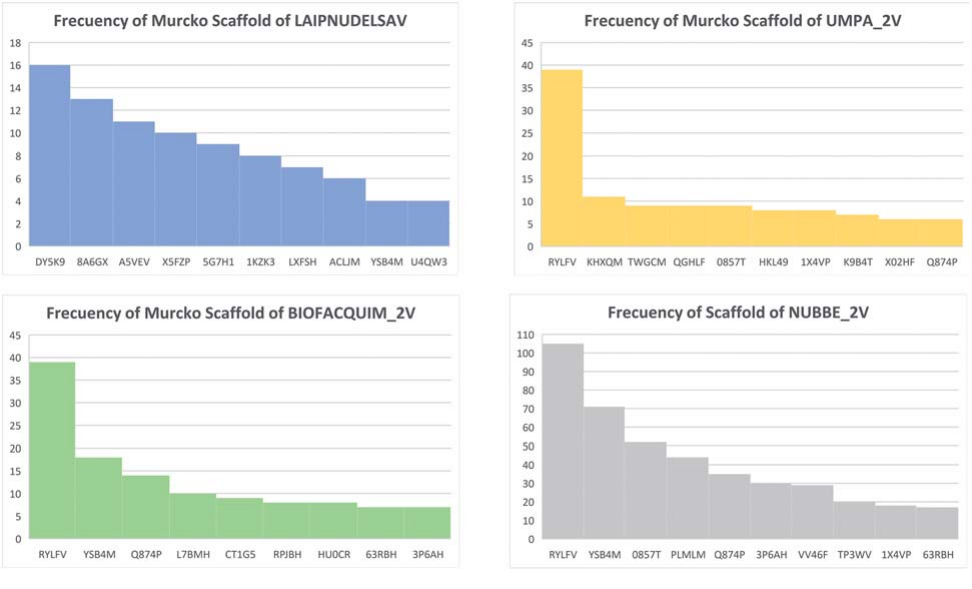
Distribution of scaffolds in LATAM_DBS_NPs.

**Fig. 8:**
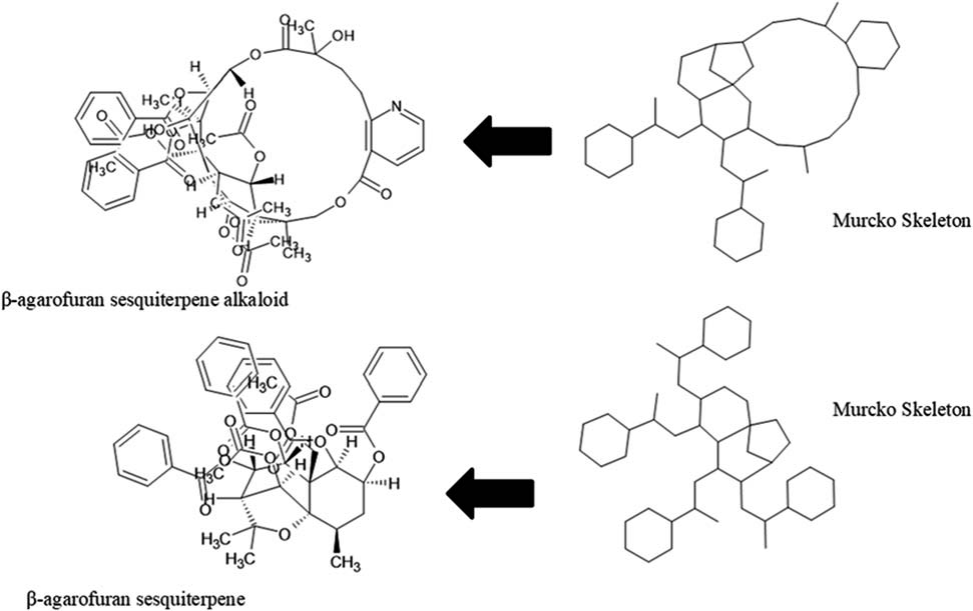
Murcko skeleton of β-agarofuran sesquiterpenoids found in LAIPNUDELSAV databases.

The content of the scaffold in LATAM_NPs has a great structural variety. The chemotypes: DY5K9, 8A6GX, A5VEV and 0857T are exclusively found in LAIPNUDELSAV_DBs. They possess the pentacyclic triterpene skeleton as shown in Fig. 5. In contrast, Fig. 6 shows some of the similar scaffolds present in LATAM_DBs: YSB4M and 1X4VP that have a benzopyran-4-one. However, these skeletons are contained in other chemotypes such as RPJBH, 63RBH, and KHXQM, which have a low frequency in BIOFACQUIM and UPMA_2V, respectively. Other chemotypes present is benzopyran-2-one identified by the codes are Q874P, 3P6AH and X02HF that are shown in Fig. 6. These nuclei are present in CT1G5, QGHLF and PLMLM, identified in the databases BIOFACQUIM, NUBBE_2V and UPMA_2V. This group constitutes the most abundant compounds in LATAM_NPs, which are shown in Fig. 7. Murcko skeletons included in the four Latin American natural product databases are similar to the nuclei present in Fig. 5 and 6. However, the β-agarofuran skeletons: X5FZP, 5G7H1, 1KZK3, LXFSH and ACLIM represent the second group of compounds in LAINPUDELSAV DBs. Fig. 8 shows two beta-agarofurans present in high frequency in the databases of the Natural Products Laboratory of the University of El Salvador, we observe their high structural complexity.

### Molecular complexity

The molecular complexity of LATAM_DBS_NPs and eleven reference databases were calculated by means of five metrics of molecular properties: a fraction of sp^3^ hybridized carbons (Fsp^3^), fraction of chiral carbon (FCC), fraction of atoms aromatic (Fa_Ar), fraction of aromatic bond (Far_b), globularity molecular (glob_m), flexibility in molecular and shape index.

**Fig. 9:**
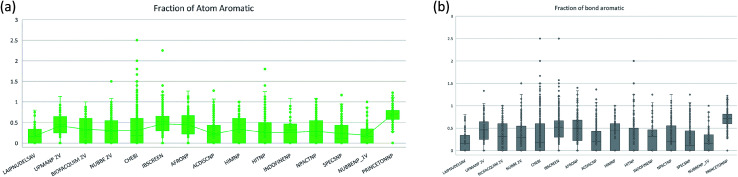
Distribution of the fraction of atoms aromatics and bond aromatic.

**Fig. 10:**
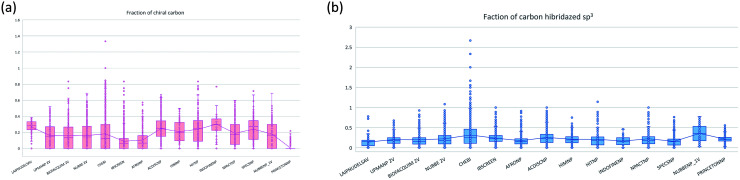
Distribution of the fraction of chiral carbon and fraction carbons hybridized sp^3^.

Fig. 9 and 10 show box plots for the distributions of Fa_aro, F_sp^3^, Fb_aro, and FCC. The globularity molecular, molecular flexibility and shape index (ESI†). Tables 7–10 summarize the statistics of Fa_aro, Fb_aro, F_sp^3^, and FCC.

**Table 7:** Metric of fraction atoms aromatics^a^

Fa_aro	Min	1Qst	Median	3Qst	Max	Mean	SD
LAIPNUDELSAV	0.00	0.00	0.15	0.33	0.80	0.17	0.19
UPMANP 2V	0.00	0.25	0.44	0.64	1.13	0.41	0.27
BIOFACQUIM_2V	0.00	0.00	0.31	0.60	1.00	0.33	0.29
NUBBE_2V	0.00	0.00	0.28	0.54	1.50	0.30	0.28
CHEBI	0.00	0.00	0.18	0.60	2.50	0.30	0.34
IBSCREEN	0.00	0.30	0.51	0.65	2.25	0.46	0.25
AFRONP	0.00	0.22	0.50	0.66	1.26	0.44	0.28
ACDISCNP	0.00	0.00	0.20	0.42	1.27	0.22	0.24
HIMNP	0.00	0.00	0.44	0.60	1.00	0.33	0.30
HITNP	0.00	0.00	0.00	0.50	1.80	0.25	0.31
INDOFINENP	0.00	0.00	0.31	0.45	1.08	0.25	0.28
NPACTNP	0.00	0.00	0.19	0.54	1.08	0.28	0.29
SPECSNP	0.00	0.00	0.11	0.42	1.16	0.23	0.26
NUBBE_1V	0.00	0.00	0.15	0.33	1.00	0.19	0.23
PRINCENTONNP	0.00	0.57	0.70	0.79	1.22	0.69	0.15

a Min: minimum; Max: maximum; Q: quartile; SD: standard deviation.

**Table 8:** Metric of fraction of bond aromatic^a^

Fb_aro	Min	1Qst	Median	3Qst	Max	Mean	SD
LAIPNUDELSAV	0.00	0.00	0.15	0.33	0.80	0.17	0.19
UPMANP 2V	0.00	0.25	0.46	0.64	1.33	0.42	0.27
BIOFACQUIM_2V	0.00	0.00	0.31	0.60	1.00	0.34	0.30
NUBBE_2V	0.00	0.00	0.28	0.54	1.50	0.30	0.28
CHEBI	0.00	0.00	0.18	0.60	2.50	0.31	0.35
IBSCREEN	0.00	0.30	0.52	0.66	2.50	0.48	0.26
AFRONP	0.00	0.22	0.50	0.67	1.40	0.45	0.29
ACDISCNP	0.00	0.00	0.20	0.42	1.36	0.22	0.24
HIMNP	0.00	0.00	0.44	0.60	1.00	0.33	0.31
HITNP	0.00	0.00	0.00	0.50	2.00	0.26	0.32
INDOFINENP	0.00	0.00	0.31	0.45	1.25	0.26	0.29
NPACTNP	0.00	0.00	0.19	0.55	1.25	0.29	0.30
SPECSNP	0.00	0.00	0.11	0.44	1.25	0.23	0.27
NUBBE_1V	0.00	0.00	0.15	0.35	1.00	0.19	0.23
PRINCENTONNP	0.00	0.60	0.72	0.81	1.23	0.70	0.15

a Min: minimum; Max: maximum; Q: auartile; SD: standard deviation.

**Table 9:** Distribution of the fraction of chiral carbon^a^

F_sp^3^	Min	1Qst	Median	3Qst	Max	Mean	SD
LAIPNUDELSAV	0.00	0.05	0.15	0.19	0.78	0.14	0.11
UPMANP 2V	0.00	0.11	0.18	0.25	0.68	0.20	0.15
BIOFACQUIM_2V	0.00	0.09	0.16	0.25	0.93	0.20	0.18
NUBBE_2V	0.00	0.10	0.18	0.30	1.09	0.22	0.18
CHEBI	0.00	0.11	0.25	0.46	2.67	0.32	0.28
IBSCREEN	0.00	0.16	0.23	0.30	1.00	0.24	0.12
AFRONP	0.00	0.10	0.16	0.22	0.94	0.18	0.14
ACDISCNP	0.00	0.13	0.23	0.33	1.04	0.26	0.18
HIMNP	0.00	0.13	0.19	0.27	0.75	0.21	0.13
HITNP	0.00	0.07	0.19	0.27	1.14	0.21	0.19
INDOFINENP	0.00	0.08	0.15	0.25	0.46	0.17	0.12
NPACTNP	0.00	0.10	0.18	0.27	1.00	0.22	0.19
SPECSNP	0.00	0.07	0.15	0.22	0.76	0.16	0.12
NUBBE_1V	0.03	0.17	0.35	0.52	0.78	0.36	0.20
PRINCENTONNP	0.00	0.17	0.21	0.26	0.56	0.21	0.07

a Min: minimum; Max: maximum; Q: quartile; SD: standard deviation.

**Table 10:** Metric of fraction carbons hybridized sp^3^^a^

FCC	Min	1Qst	Median	3Qst	Max	Mean	SD
LAIPNUDELSAV	0.00	0.23	0.29	0.33	0.38	0.27	0.08
UPMANP 2V	0.00	0.00	0.15	0.27	0.52	0.16	0.15
BIOFACQUIM_2V	0.00	0.00	0.13	0.27	0.83	0.16	0.16
NUBBE_2V	0.00	0.00	0.14	0.28	0.69	0.16	0.14
CHEBI	0.00	0.00	0.13	0.30	1.33	0.18	0.20
IBSCREEN	0.00	0.00	0.06	0.13	0.83	0.09	0.10
AFRONP	0.00	0.00	0.07	0.16	0.57	0.09	0.10
ACDISCNP	0.00	0.15	0.25	0.34	0.68	0.10	0.12
HIMNP	0.00	0.10	0.20	0.32	0.50	0.21	0.14
HITNP	0.00	0.09	0.25	0.35	0.83	0.24	0.18
INDOFINENP	0.00	0.22	0.30	0.37	0.77	0.30	0.19
NPACTNP	0.00	0.05	0.17	0.30	0.60	0.19	0.15
SPECSNP	0.00	0.11	0.27	0.35	0.71	0.24	0.14
NUBBE_1V	0.00	0.00	0.17	0.30	0.69	0.18	0.16
PRINCENTONNP	0.00	0.00	0.00	0.00	0.23	0.01	0.02

a Min: minimum; Max: maximum; Q: quartile; SD: standard deviation.

The analysis of the fraction of atoms and bond aromatics having this database shows median values between 0.44 and 0.71 for PRINCETON_NP, IBSCREEN_NP, HIM_NP, AFRONP and UPMA_NP_2V, indicating high aromaticity and rigidity in these databases. However, the analysis of the carbon fraction with sp^3^ hybridization indicates that the databases IBSCREEN_NP, CHEBI, ACDISC_NP, PRINCETON_NP and NUBBE_1V are the most complex, showing values from 0.22 to 0.35, while LAIPNUDELSAV, AFRO_NP, BIOFACQUIM_2V INDOFINE_NP, UPMA_NP 2V, and NPACT_NP are the least complex with values between 0.15 and 0.17. Otherwise, the chiral carbon fraction indicates that HIM_NP, HIT_NP, ACDIS_NP, SPECS_NP, LAIPNUDELSAV and INDOFINE_NP have a greater number of chiral carbons with values of 0.20 to 0.30.

The analysis of the molecular flexibility and the shape index of LATAM_DBS_NPs and REF_NP suggests that the compounds have high structural rigidity and spherical shape (non-linear) since they present values less than 0.50 for most of the analyzed databases (ESI†).

The globularity molecular is not a good metric of complexity because it does not differentiate the data sets analyzed in this work (ESI†).

### Molecular docking

#### Protein selection

SARS-CoV-2 is a virus that has four structural proteins and sixteen nonstructural proteins, that are essential for its replication cycle. Structural proteins include: spike, membrane, nucleocapsid and envelope. Nonstructural proteins (NSP) of the CoV include NSP-1 protein to NSP-16 protein with the functions of cutting, splitting and joining RNA. The NSP-5 has the protease types 3CLpro and Mpro while NSP-9 is an RNA replicase. Meanwhile, NSP-12 is an RNA dependent RNA polymerase, NSP-13 is a helicase, NSP-14 is an exonuclease, NSP-15 is an endoribonuclease and NSP-16 is a 2′O-methyltransferase.^51^

NSP-15 encodes a uridylate-specific endoribonuclease enzyme (EndoU), indispensable for the viral cycle of coronavirus (COVID-19). This facilitates the breakdown of RNA at the ends of the uridylates. The loss of NSP-15 directly impacts RNA replication processes and their pathogenesis. Therefore, NSP-15 constitutes a potential therapeutic target for the development of inhibitors of endoribonuclease-dependent viral replication.^52^

NSP-15 (endoribonuclease) was selected for this work since there is little research on this target of the coronavirus (SARS-CoV-2).

The structure NSP-15 (endoribonuclease) complex with tipiracid was obtained from the Protein Data Bank. This viral protein of the severe acute respiratory syndrome coronavirus 2 (SARS CoV-2) was encoded as 6WXC PDB and possesses uridylate-specific endoribonuclease with A and B chains.^53^

#### Validation of docking molecular protocol with endoribonuclease-NSP-15

**Fig. 11:**
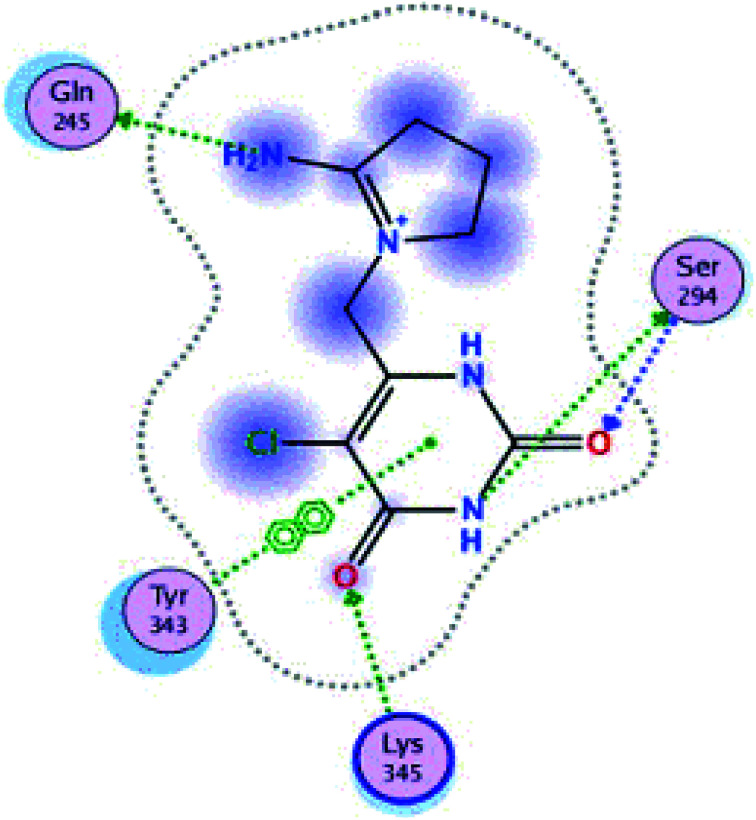
Interaction of ligand co-crystallized CMU in site active of endoribonucleases NSP-15.

The docking protocol was validated by re-docking, we used the viral protein (ID 6WXC) with the structure of the co-crystallized compounds (tipiracid = CMU: 5-chloro-6-(1-2-iminopyrrolidinyl)-methyl-uracil). This was subjected to a simulated auto mode coupling-induced fit. In this study, we observed good reproducibility of the co-crystallized inhibitor confirmation with an RMSD value of 2.8021 and a relative docking score for the ligand (CMU) of −5.4824. The binding pocket was defined as the set of amino acids within 1.85 Å of the co-crystallized ligand CMU. This active site of endoribonuclease in the model of 2D is shown in Fig. 11 (6WXC ID PDB) and has residues of amino acids, Gln245, Ser294, and Lys345 that show four hydrogen bonds and one pi–pi interaction with Tyr343 with CMU, that possess calculated binding energy of 27.2 kcal mol^−1^ (MOE Dock Panel).

#### Simulation of docking

For the docking analysis, we selected riboendonuclease NSP-15 (viral protein of SARS CoV-2) and evaluated 130 compounds of de LAIPNUDELSAV and 352 compounds of UPMA_2V DB. These databases LAIPNUDELSAV and UPMA_NP_2V were selected by taking results of analysis cheminformatics, which indicates that these two databases are more diverse based on fingerprint and Tanimoto similarity index Cosine. Further, this group of compounds was selected using the following criteria of restriction, scaffold diversity, molecular complexity, and Lipinski rules.

**Fig. 12:**
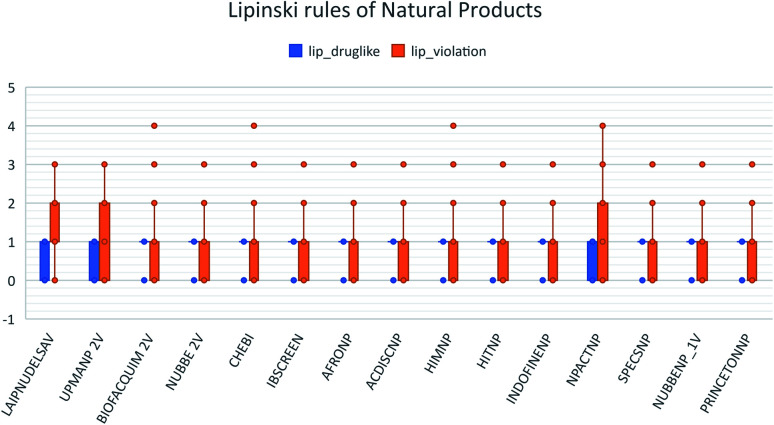
Lipinski rules of natural products analysed in this work.

**Fig. 13:**
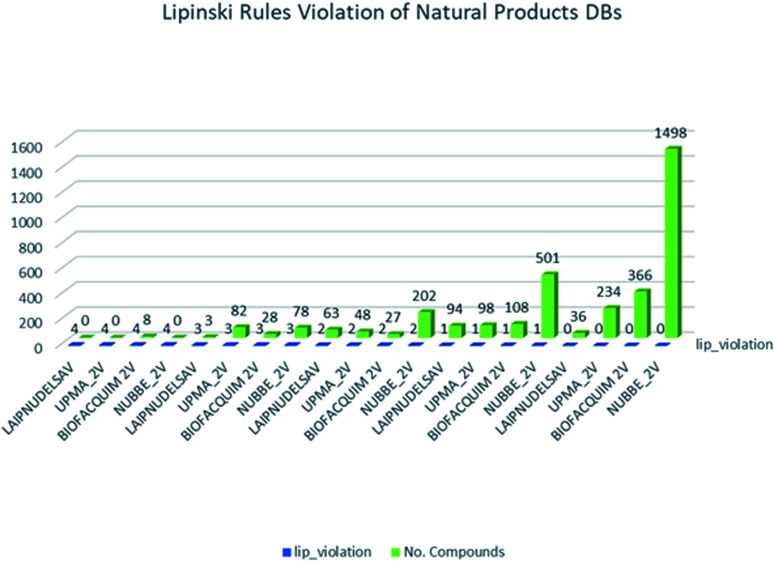
Distribution of natural products for Lipinski rules.

**Fig. 14:**
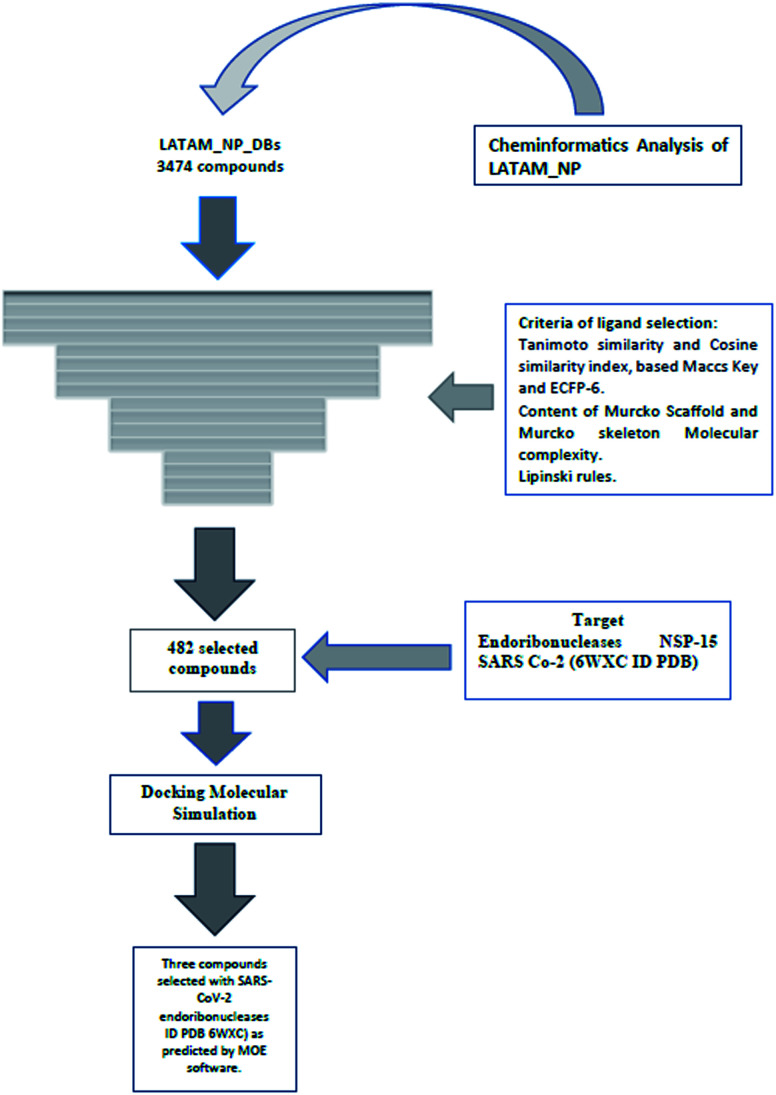
The workflow utilized in this work.

These data indicate that LAIPNUDELSAV and UMPA_2V are the two most diverse, have more fraction contents of Murcko scaffold and Murcko skeleton, and overly complex ring systems. These 482 compounds were selected using inclusion criteria, one violation of the Lipinski rules. Fig. 12 shows the comparison of drug-like properties and the violation of Lipinski's rule for the fifteen databases of natural products evaluated, meanwhile, Fig. 13 presents an illustration of distribution by the number of compounds in all databases of natural products that meet and break the Lipinski rule (Fig. 14).

**Fig. 15:**
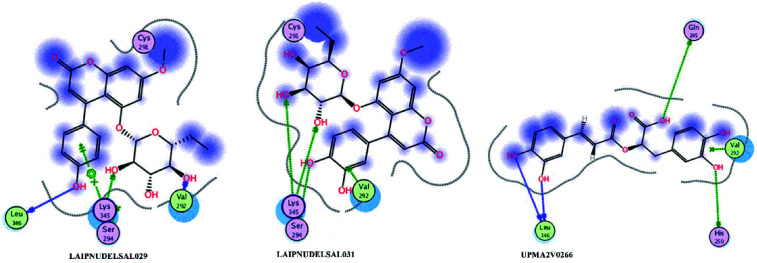
Binding interaction of NPs in site active of endoribonucleases NSP-15 on 2D.

**Fig. 16:**
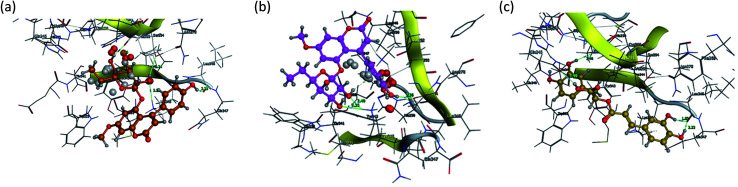
Binding interaction of LAIPNUDELSAV_029, LAIPNUDELSAV_031 and UPMA_2V_0266 within the active site of endoribonucleases NSP-15 in 3D.

Fig. 15 and 16 visualize the binding mode between the ligands, LAIPNUDELSAV_029, LAIPNUDELSAL_031 and UPMA_2V_0266 in the active site of endoribonucleases NSP-15 in model 2D and 3D.

LAIPNUDELSAV_029 and LAIPNUDELSAV_031 corresponding a 4-phenylcoumarins with O-glycosidic bonds in C-7 and the hydroxyl in C-1′ of sugar residues,^54^ while UPMA-2V_0266 a derivative of caffeic acid.^55^

These three compounds share with the cocrystallized ligand (CMU) the hydrogen bridge interaction with Ser294A and Lys345A residues, while we observed additional interactions with Val292, Leu346 and His 250 residues with these natural products.

Phenylcoumarins and rosmarinic acid interact in a similar way at the binding site of the NSP-15 protein but occupy a greater volume at this site, therefore, we propose that these substances could act as inhibitors of this viral protein, but these statements must be validated in subsequent studies of this work.

Table 11 shows the functional groups of the three ligands that interact with amino acid residues in the active site of NSP-15. In addition, it includes the docking score, RMSD, and binding energy values obtained from the molecular docking simulation with the MOE v2019.01 software.^49^

**Table 11:** Molecular docking of select drugs onto the SARS-CoV-2 Nsp15 endoribonuclease

Ligand group	Receptor	Residue of amino acids	Types of interaction	Energy of bond interaction, kcal mol^−1^	Docking score	RMSD refine	Binding energy, kcal mol^−1^
**LAIPNUDELSAV_029**					**−5.1983**	**2.1983**	**−10.1**
H–O–C-4′	O	LEU346 A	H-Donor	−1.7			
H–O–C-3′′	O	SER294 A	H-Donor	−2.3			
H–O–C-5′′	O	VAL292 A	H-Donor	−3.0			
H–O–C-3′′	N	LYS345 A	H-Acceptor	−2.4			
6-Ring	N	LYS345 A	Pi-Cation	−0.8			

**LAIPNUDELSAV_031**					**−5.0742**	**1.6492**	**−4.9**
H–O–C-4′′	N	LYS345 A	H-Acceptor	−1.7			
H–O–C-3′′	O	LYS345 A	H-Acceptor	−1.3			
H–O–C-5′′	O	SER294 A	H-Donor	−1.7			
6-Ring	N	VAL292 A	Pi-Cation	−0.2			

**UPMA-2V_0266**					**−5.7371**	**2.6559**	**−8.7**
H–O–C-14	N	HIS250 A	H-Donor	−2.1			
H–O–C=O (C-18)	O	GLN245 A	H-Donor	−1.4			
H–O–C-4	O	LEU346 A	H-Donor	−1.3			
H–O–C-3	O	LEU346 A	H-Donor	−3.7			
6-Ring	N	VAL292 A	Pi-Cation	−0.2			

**TIPIRACID (CMU)**					**−5.4824**	**1.7440**	**−27.2**

These skeletons have biological activity against HIV, and tuberculosis, and has antioxidant activity.

The docking study has allowed the identification of two 4-phenylcoumarins and catechol derivatives as potential inhibitors of riboendonuclease NPS-15 of SARS-CoV-2. A variety of NPs (alkaloid, catechol derivative, benzofuran, benzopyran, polyphenols)^56^ and 4-methyl-coumarin derivatives^57^ have been reported as inhibitors of structural and no structural proteins of COVID-19.

## Conclusions

In this cheminformatics study, we analysed 46 413 compounds from four unique NPs from Latin American natural products and eleven published NPs databases used as references. These NPs occupy similar chemical spaces, therefore share properties of interest in the bioprospecting project of NPs for the discovery and development of lead molecules of therapeutic potential.

The databases of the University of El Salvador and the University of Panama present the greatest structural diversities based on the fingerprint. However, when comparing the chemotype relationship, NPs of Africa, Mexico, and Panama have the highest scaffold diversity. The University of El Salvador database, herbal ingredients active targets, and ICC indofine present the greatest diversity in the Murcko skeleton content bases at *F*_50_. The content of the scaffold in three LATAM_NPs predominated compound of types benzopyran-one with modification in positions 2 and 4 of this cyclic system with anti-radical and antioxidant effects, meanwhile pentacyclic triterpenes and β-agarofuran prevailed in LAIPNUDELSAV. This last group of compounds has a reported an anti-inflammatory effect by inhibiting NOS.

From the analysis of molecular complexity, it is concluded that the compounds of the University of Panama are the ones with the highest aromaticity and structural rigidity. The opposite case occurs with NUBBE_1V_2013, which has a large fraction of sp3 carbons, while the databases of the University of El Salvador, Mexico, and Panama have fewer types of carbons; additionally, the LAIPNUDELSAV contains the highest number of chiral carbons. By contrast, the comparison of the molecular flexibility and the shape index of the Latin American NPs and the reference NPs show that all evaluated compounds have high structural rigidity.

Molecular docking simulation has allowed identification of 5-*O*-β-d-glucopyranosyl-4′-hydroxy-7-methoxy-4-phenylcoumarin, 5-*O*-β-d-galactopyranosyl-3′,4′-dihydroxy-7-methoxy-4-phenylcoumarin and rosmarinic acid as potential inhibitors of riboendonuclease NPS-15 of SARS-CoV-2. This is the first in the silico study evaluating the potential of Latin American natural products against COVID-19. Furthermore, this work can contribute to helping in the future design of new candidate anti-SARS-CoV-2.

## Author contributions

M. J. N. participated in the conceptualization, design, and creation of databases of the University of El Salvador. He was also actively involved in the analysis of results, writing, reviewing, and editing of the manuscript. B. I. D. E. T. was involved in data curation, computational analysis, designing computer program, supporting algorithms and validation of the results. She also collaborated in the writing, review, and edition of the manuscript. J. L. M. F. has worked in the conceptualization of research, review of methodological design, analysis, visualization, and statistical analysis of the results. Also, he participated in the writing, review, and edition of the manuscript. D. A. O. A. participated in the conceptualization, creation, conducting, methodological design of the research and obtaining funds. He also actively collaborated on data curation, statistical evaluation, computational analysis, writing, reviewed editing manuscript.

## Conflicts of interest

Authors declare that there are no financial or commercial conflicts of interest.

## Supplementary Material

RA-011-D1RA01507A-s001
