# Supplementary material for: Latin American databases of natural products: biodiversity and drug discovery against SARS-CoV-2
Source: RSC Adv. 2021 May 4;11(26):16051–64. doi: 10.1039/d1ra01507a (PMC9030473; doi:10.1039/d1ra01507a)
Supplement: RA-011-D1RA01507A-s001 [file RA-011-D1RA01507A-s001.pdf]

Electronic Supplementary Material (ESI) for RSC Advances.
This journal is © The Royal Society of Chemistry 2021

Electronic supplementary information (ESI)

Latin American Databases of Natural Products: Biodiversity and Drug Discovery against SARS-CoV-2.

Marvin J. Nuñez[a], Bárbara I. Díaz-Eufracio[b], José L. Medina-Franco[b] and Dionisio A. Olmedo[c,d]*

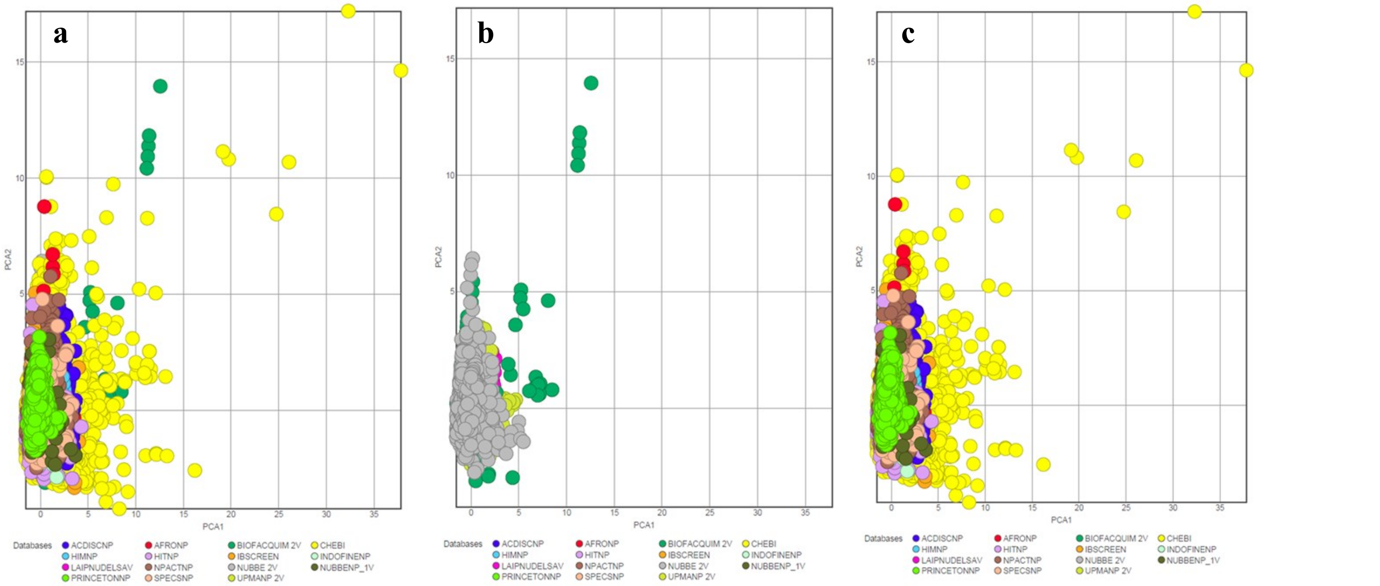

Figure S1 *Representation of the chemical space in 2D. All NPs (**a**), LATAM_DBS_NPs (**b**) and REF_NPs (**c**).*

Table S1. *Summary of Principal Component Analysis (PCA) of s ix physicochemical properties (PCP)*

PCP	PCA	Deviation	Condition	% Var
**S log P**	1	2.00036156e+000	1.00000000e+000	66.691
**TPSA**	2	1.15902870e+000	2.97871271e+000	89.080
**Weight**	3	6.13420952e-001	1.06340767e+001	95.351
**a_acc**	4	4.28659458e-001	2.17767056e+001	98.414
**a_don**	5	2.60168406e-001	5.91163876e+001	99.542
**b_rotN**	6	1.65783895e-001	1.45590261e+002	100.000

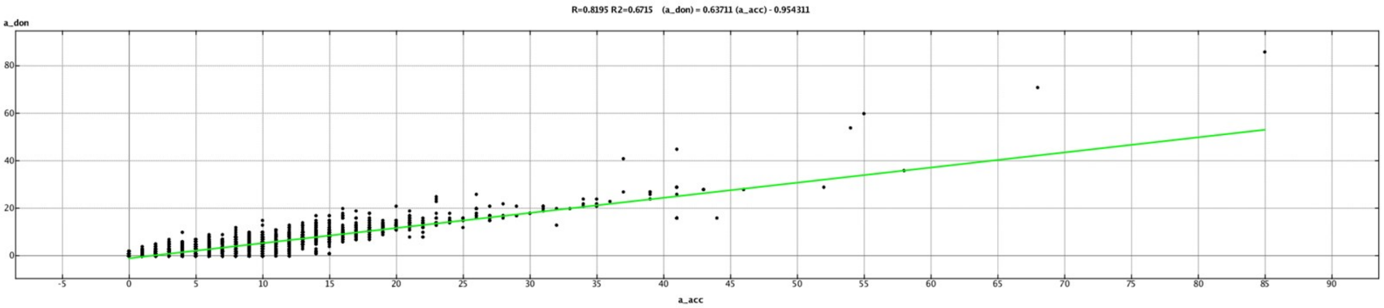

Figure S2 *Correlation scatter plot hydrogen bond acceptors (HBAs), and hydrogen bond donors (HBDs) in DBs*

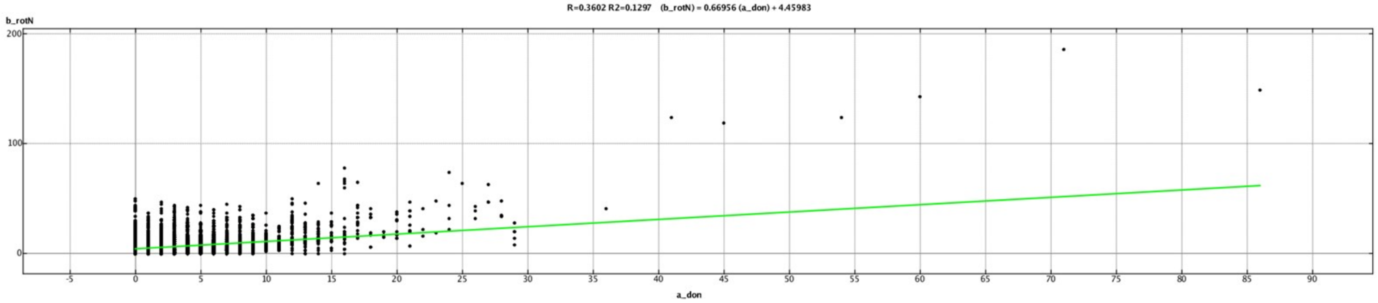

Figure S3 *Correlation scatter plot hydrogen bond donors and number of rotatable bonds (NRBs) in data set.*

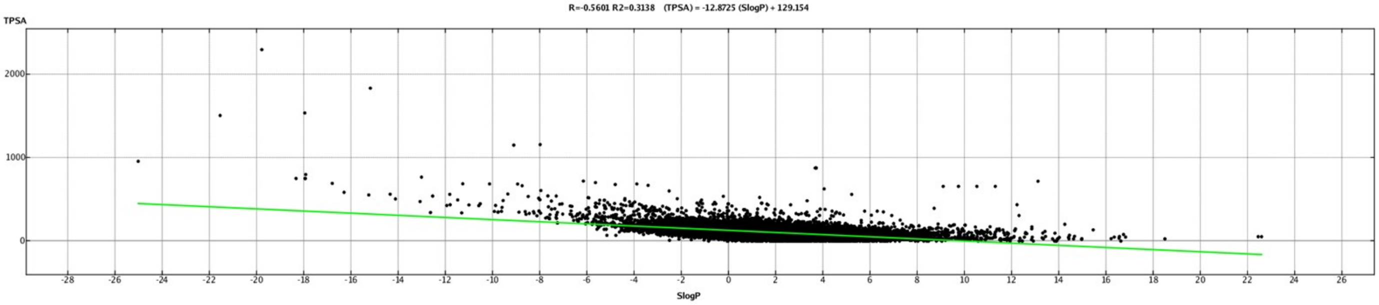

Figure S4 *Correlation scatter plot S Log P and TPSA in DBs*

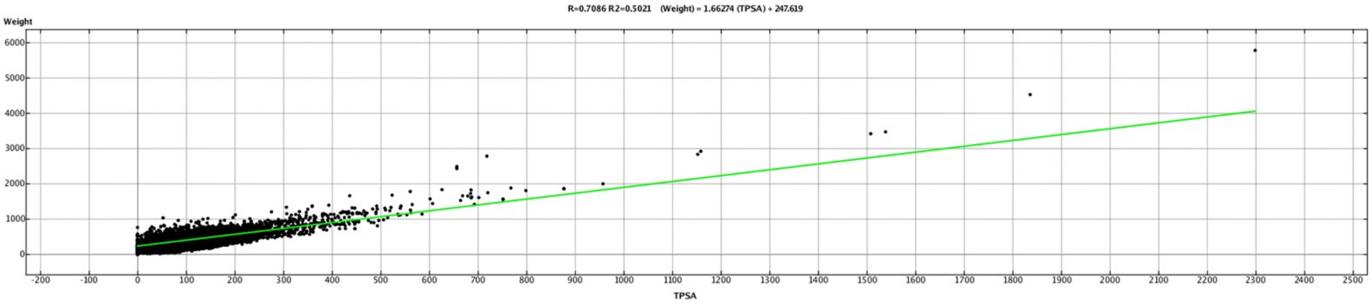

Figure S5 *Correlation scatter plot TPSA and Weight Molecular in DBs*

Figure S1-S5 *Correlation Scatter Plot of Physicochemical properties of the databases.* ***Note:*** *These correlation scatter graphs show that the groups of hydrogen acceptors / donors, the partition coefficient / TPSA, were adjusted to the regression model, observing a great association between these physicochemical properties of therapeutic interest.*

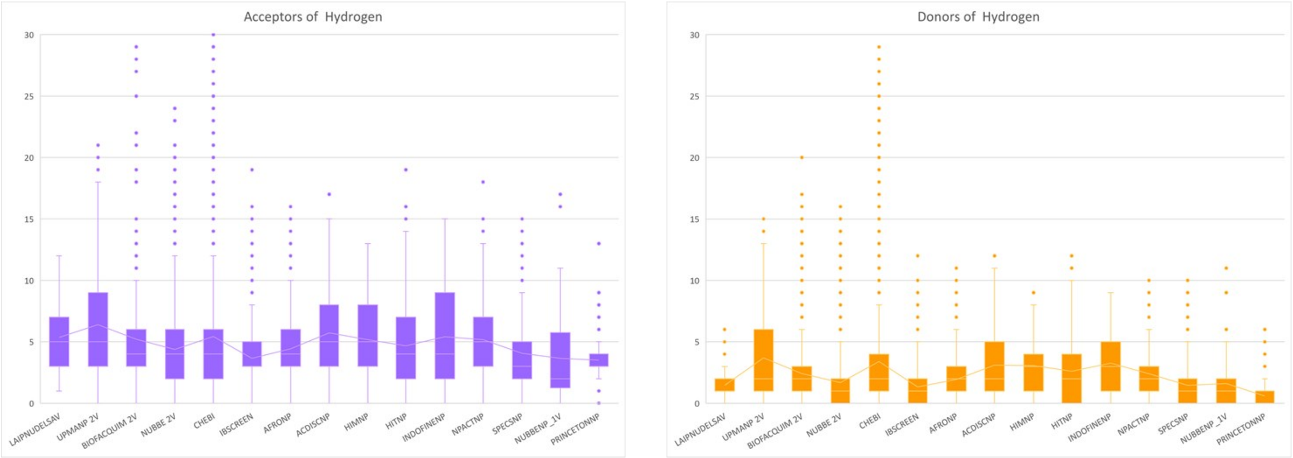

Figure S6 *Box plot of hydrogen bond acceptors (HBAs), hydrogen bond donors (HBDs) in Dbs.*

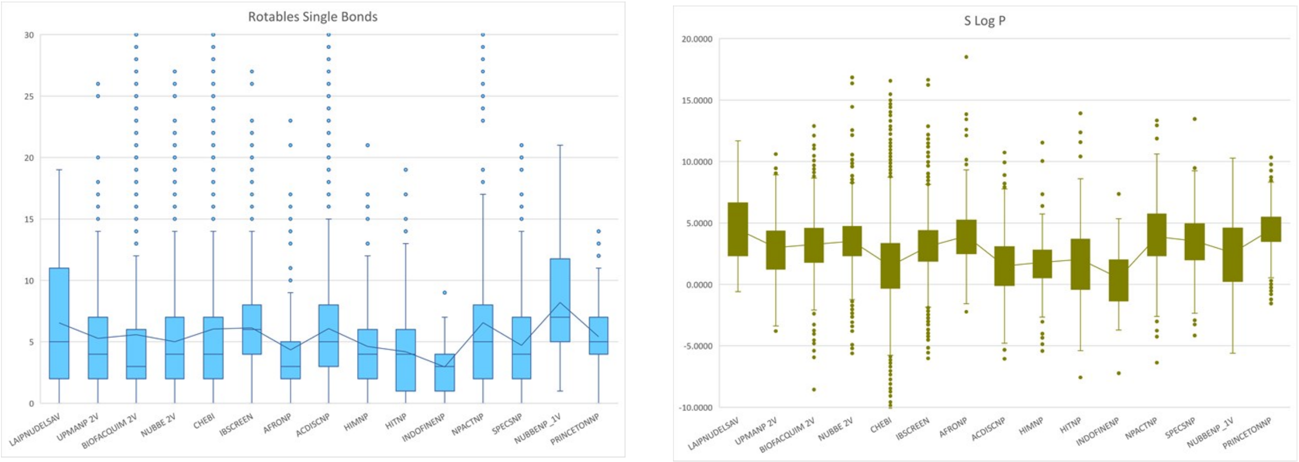

Figure S7 *Box plot of rotatable bonds (NRBs) and the octanol/water partition coefficient (w/o; S Log P) in databases*

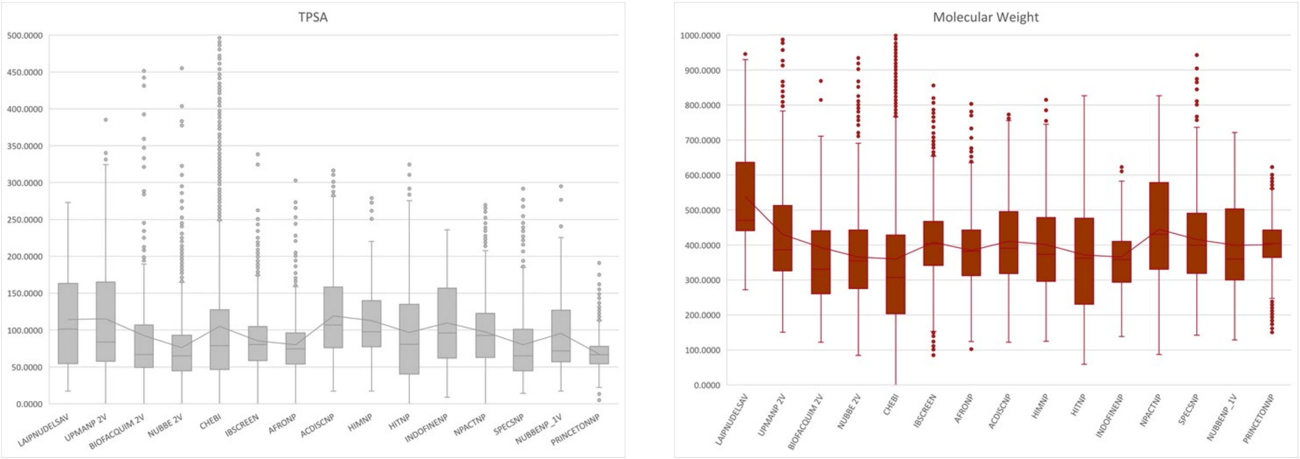

Figure S8 *Box plot of topological polar surface area (TPSA and molecular weight (MW)in dataset.*

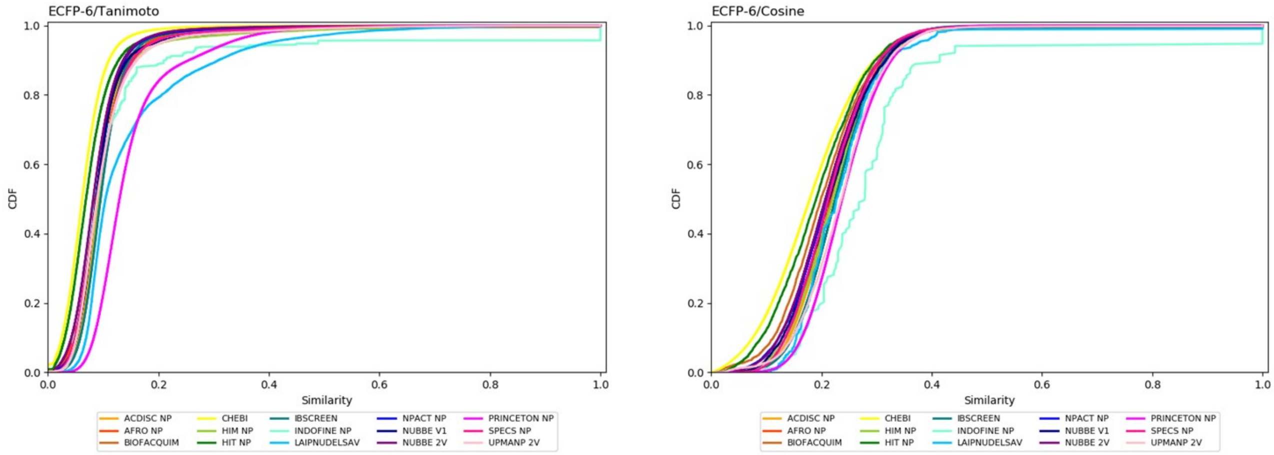

Figure S9 *Cumulative distribution function of the pairwise-similarity of the different data sets computed with ECFP-6-Tanimoto and Cosine.*

Table S2 *Summary statistics of the pairwise similarity computed with Tanimoto-ECFP-6*

**NPs_DBs**	**mean**	**SD**	**min**	1st Q	**median**	**3^{rd}Q**	**max**
**ACDISCNP**	0.0998	0.0504	0.0	0.0714	0.0909	0.1154	1.0
**AFRONP**	0.0966	0.0558	0.0	0.0641	0.0873	0.1167	1.0
BIOFACQUIM	0.0944	0.0567	0.0	0.0654	0.0862	0.1104	1.0
**CHEBI**	0.6726	0.0413	0.0	0.0421	0.0625	0.0849	1.0
**HIM_NP**	0.1079	0.0925	0.0	0.0682	0.0903	0.1176	1.0
**HIT_NP**	0.0809	0.7522	0.0	0.0476	0.0691	0.0948	1.0
**IBSCREEN**	0.1026	0.0494	0.0	0.0753	0.0955	119	1.0
**INDOFINE NP**	0.1385	0.1942	0.0108	0.0614	0.0821	0.1259	1.0
LAIPNUDELSAV	0.1526	0.1207	0.0161	0.0824	0.1043	0.1718	1.0
**NPACT NP**	0.0960	0.0590	0.0	0.0677	0.0879	0.1103	1.0
**NUBBE 1V**	0.1010	0.0717	0.0	0.0685	0.0876	0.1111	1.0
**NUBBE 2V**	0.0893	0.0517	0.0	0.0610	0.0822	0.1068	1.0
**PRINCETON NP**	0.1532	0.0815	0.0099	0.1045	0.1314	0.1698	1.0
**SPECSNP**	0.1017	0.0704	0.0	0.0657	0.0889	0.1189	1.0
UPMANP 2V	0.1051	0.0747	0.0	0.0676	0.0893	0.1189	1.0

Min: Minimum; Max: Maximum; Q: Quartile; SD, Standard Deviation.

Table S3 *Summary statistics of the pairwise similarity computed with Cosine-ECFP-6*

NPS_DBS	mean	SD	min	1st Q	median	3rdQ	max
**ACDISC NP**	0.2252	0.0694	0.0143	0.1766	0.2201	0.2680	1.0
**AFRO NP**	0.2226	0.0775	0.0129	0.1719	0.2178	0.2651	1.0
BIOFACQUIM	0.2061	0.0878	0.0022	0.1518	0.1990	0.2524	1.0
CHEBI	0.1084	0.0865	0.0001	0.1232	0.1790	0.2587	1.0
**HIM NP**	0.2274	0.0935	0.0328	0.1740	0.2201	0.2690	1.0
**HIT NP**	0.1970	0.1000	0.0122	0.1358	0.1904	0.2472	1.0
**IBSCREEN**	0.2288	0.0665	0.0118	0.1832	0.2253	0.2707	1.0
**INDOFINE NP**	0.3052	0.1889	0.0615	0.2045	0.2789	0.3142	1.0
LAIPNUDELSAV	0.2406	0.1032	0.1070	0.1823	0.2280	0.2732	1.0
**NPACTNP**	0.2106	0.0768	0.0123	0.1606	0.2080	0.2559	1.0
**NUBBE 1V**	0.2195	0.0808	0.0387	0.1677	0.2125	0.2622	1.0
NUBBE 2V	0.2123	0.0756	0.0079	0.1613	0.2072	0.2580	1.0
**PRINCETON NP**	0.2415	0.0637	0.0298	0.1962	0.2365	0.2813	1.0
**SPECSNP**	0.2182	0.0787	0.0431	0.1688	0.2122	0.2581	1.0
UPMANP 2V	0.2364	0.0805	0.0071	0.1881	0.2363	0.2790	1.0

Min: Minimum; Max: Maximum; Q: Quartile; SD, Standard Deviation.

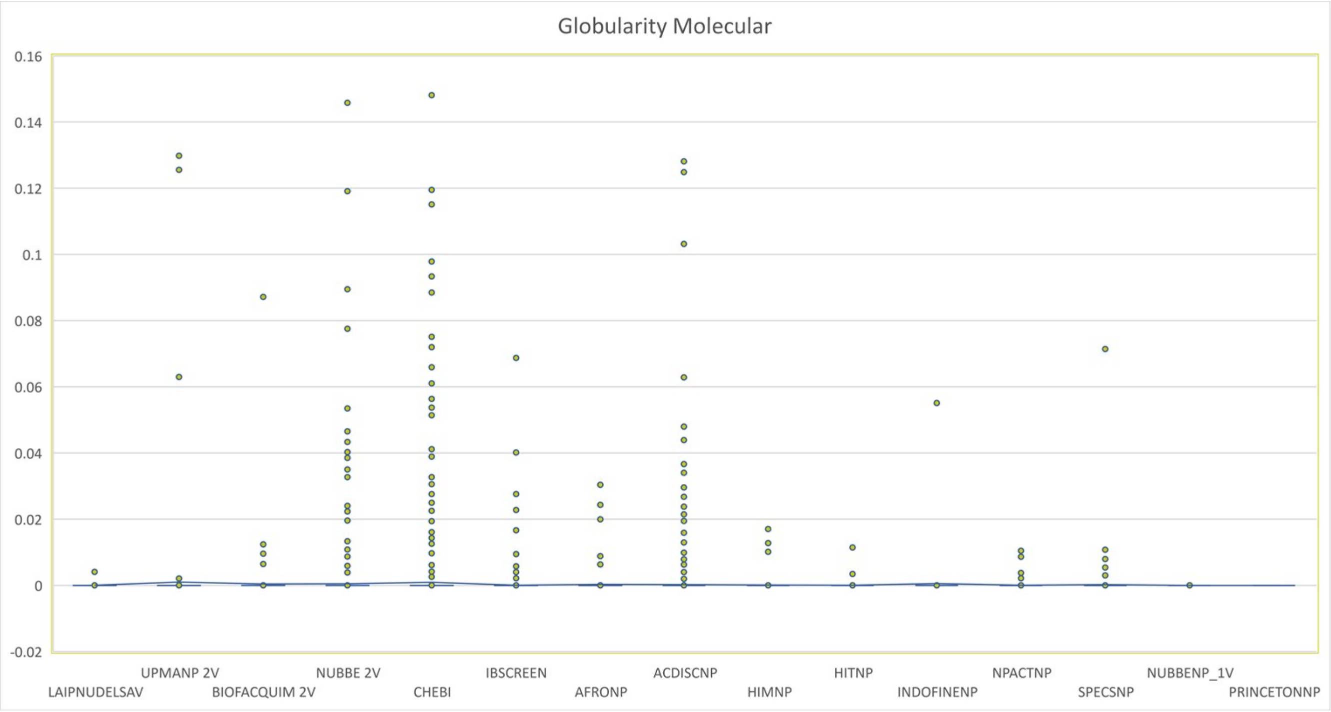

Figure S10 *Distribution of globularity molecular*

Table S4 *Metric of globularity molecular*

glob_mol	Mean	SD
**LAIPNUDELSAV**	0.0000	0.0004
**UPMANP 2V**	0.0010	0.0107
**BIOFACQUIM_2V**	0.0004	0.0055
**NUBBE_2V**	0.0005	0.0056
**CHEBI**	0.0009	0.0238
**IBSCREEN**	0.0000	0.0010
**AFRONP**	0.0003	0.0022
**ACDISCNP**	0.0002	0.0030
**HIMNP**	0.0001	0.0013
**HITNP**	0.0000	0.0007
**INDOFINENP**	0.0006	0.0055
**NPACTNP**	0.0001	0.0007
**SPECSNP**	0.0002	0.0029
**NUBBE_1V**	0.0000	0.0000
**PRINCENTONNP**	0.0000	0.0000

Mean; SD: Standard Deviation.

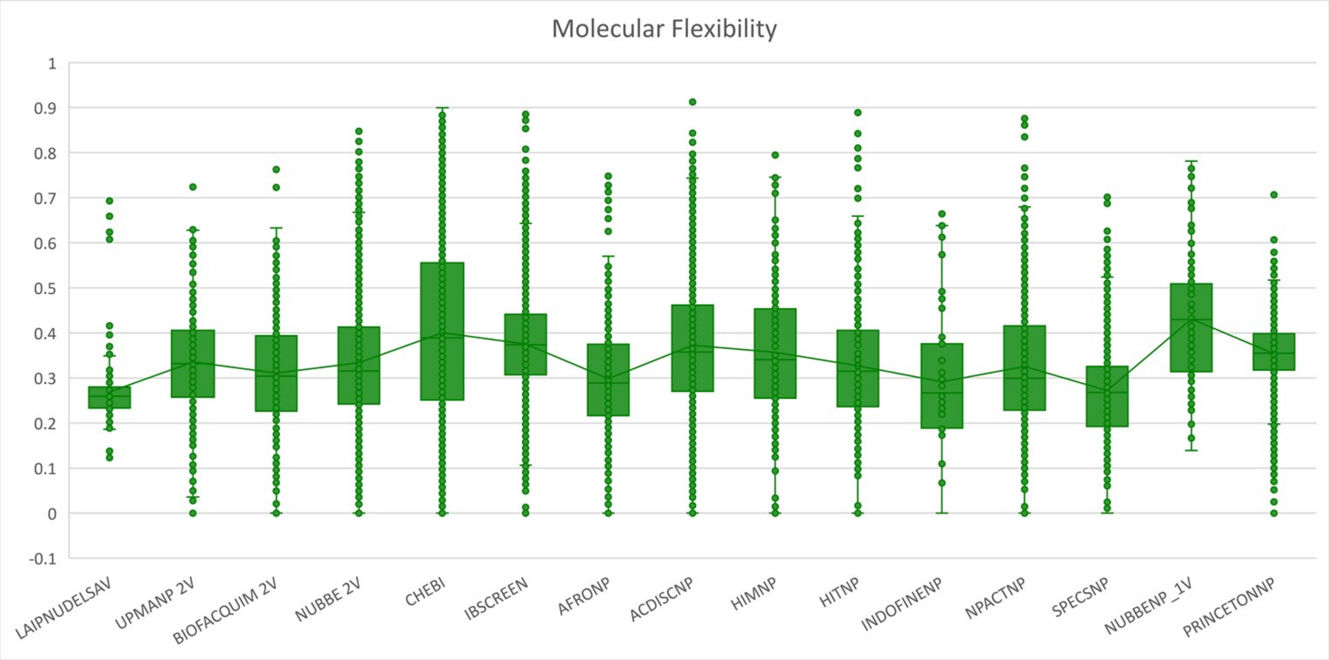

Figure S11. *Distribution of molecular flexibility*

Table S6. *Metric of flexibility molecular*

Molecular Flexibility	Min	1Qst	median	3Qst	Max	Mean	SD
**LAIPNUDELSAV**	0.1230	0.2336	0.2593	0.2790	0.6952	0.2688	0.0786
**UPMANP 2V**	0.0000	0.2582	0.3320	0.4055	0.7242	0.3354	0.1276
**BIOFACQUIM_2V**	0.0000	0.2268	0.3042	0.3934	0.7626	0.3110	0.1330
**NUBBE_2V**	0.0000	0.2423	0.3157	0.4124	0.8518	0.3339	0.1407
**CHEBI**	0.0000	0.2512	0.3888	0.5554	0.8999	0.4007	0.2058
**IBSCREEN**	-1.0000	0.3071	0.3738	0.4415	0.8854	0.3751	0.1140
**AFRONP**	0.0000	0.2172	0.2887	0.3745	0.7482	0.2982	0.1321
**ACDISCNP**	0.0000	0.2705	0.3573	0.4616	0.9126	0.3726	0.1424
**HIMNP**	0.0000	0.2559	0.3408	0.4531	0.8076	0.3569	0.1421
**HITNP**	0.0000	0.2365	0.3151	0.4051	0.8888	0.3569	0.1421
**INDOFINENP**	0.0000	0.1895	0.2671	0.3756	0.6642	0.2915	0.1235
**NPACTNP**	0.0000	0.2285	0.2995	0.4155	0.8759	0.3256	0.1439
**SPECSNP**	0.0000	0.1926	0.2680	0.3249	0.7017	0.2721	0.1062
**NUBBE_1V**	0.1389	0.3146	0.4298	0.5087	0.7813	0.4310	0.1447
**PRINCENTONNP**	0.0000	0.3177	0.3549	0.3981	0.7076	0.3536	0.0660

Min: Minimum; Max: Maximum; Q: Quartile; SD: Standard Deviation.

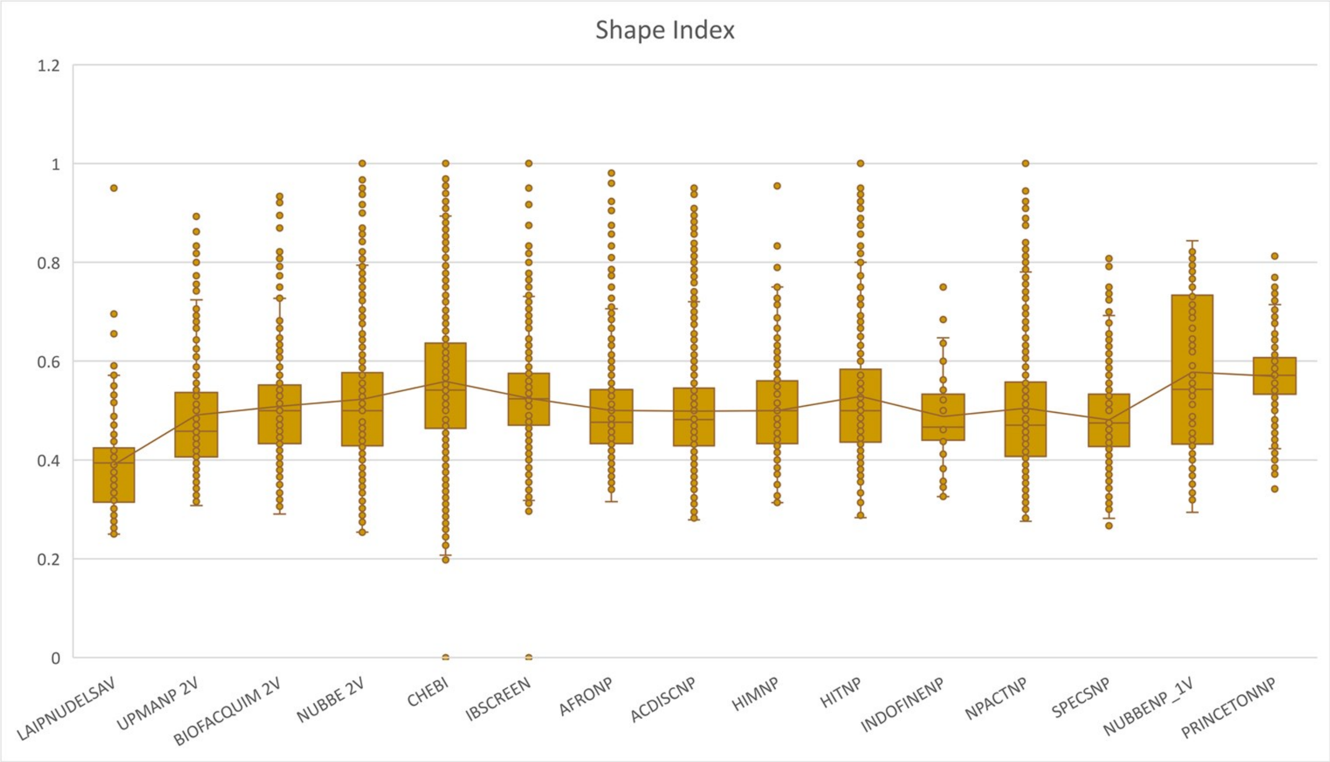

Figure S12 *Distribution of shape index*

Table S7 *Metric of shape index*

Shape Index	Min	1Qst	Median	3Qst	Max	Mean	SD
**LAIPNUDELSAV**	0.2500	0.3171	0.3939	0.4242	0.9500	0.3902	0.1009
**UPMANP 2V**	0.3077	0.4063	0.4583	0.5358	0.8929	0.4909	0.1169
**BIOFACQUIM_2V**	0.2906	0.4333	0.5000	0.5517	0.9375	0.5087	0.1238
**NUBBE_2V**	0.2537	0.4286	0.5000	0.5769	1.0000	0.5227	0.1369
**CHEBI**	0.0000	0.4643	0.5417	0.6364	1.0000	0.5592	0.1431
**IBSCREEN**	0.0000	0.4706	0.5238	0.5926	1.0000	0.5248	0.0753
**AFRONP**	0.3158	0.4333	0.4762	0.5417	0.9808	0.5005	0.1055
**ACDISCNP**	0.0000	0.4286	0.4815	0.5455	0.9583	0.4356	0.1397
**HIMNP**	0.2917	0.4333	0.5000	0.5600	0.9546	0.4994	0.0921
**HITNP**	0.2833	0.4369	0.5000	0.5833	1.0000	0.5285	0.1300
**INDOFINENP**	0.3261	0.4400	0.4667	0.5275	0.7500	0.4880	0.0877
**NPACTNP**	0.0000	0.4074	0.4706	0.5556	1.0000	0.4151	0.1663
**SPECSNP**	0.2667	0.4286	0.4750	0.5333	0.8077	0.4814	0.0818
**NUBBE_1V**	0.5417	0.5469	0.5521	0.5573	0.5625	0.5521	0.0147
**PRINCENTONNP**	0.3415	0.5333	0.5714	0.6071	0.8125	0.5693	0.0534

Min: Minimum; Max: Maximum; Q: Quartile; SD: Standard Deviation.

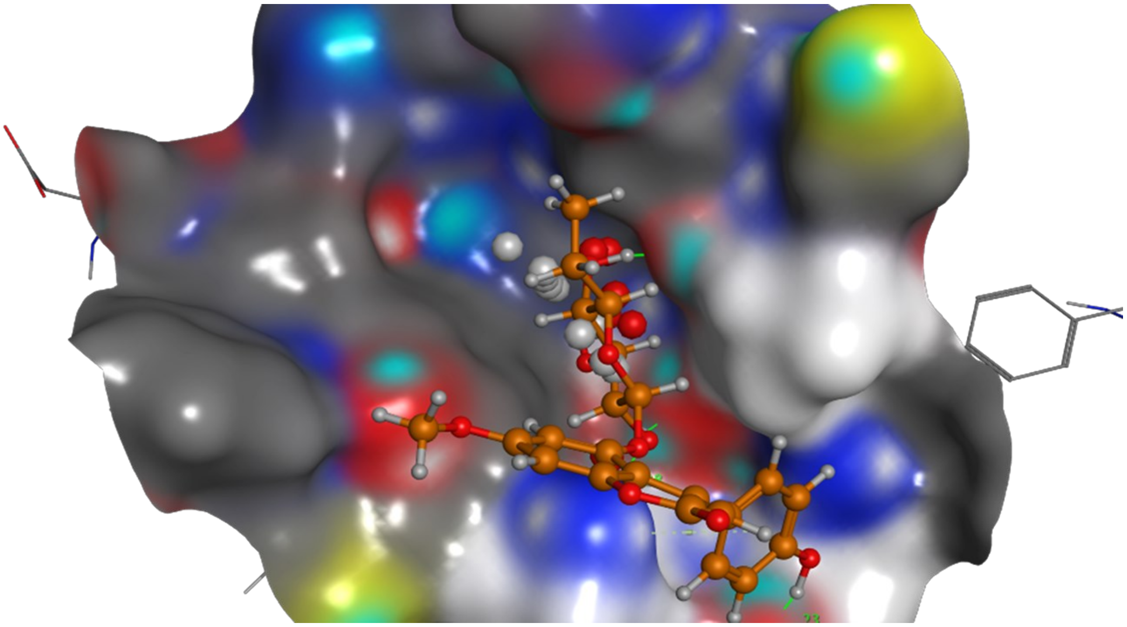

Figure S13 Ligand_LAIPNUDELSAV_29 in the pocket of protein NSP-15

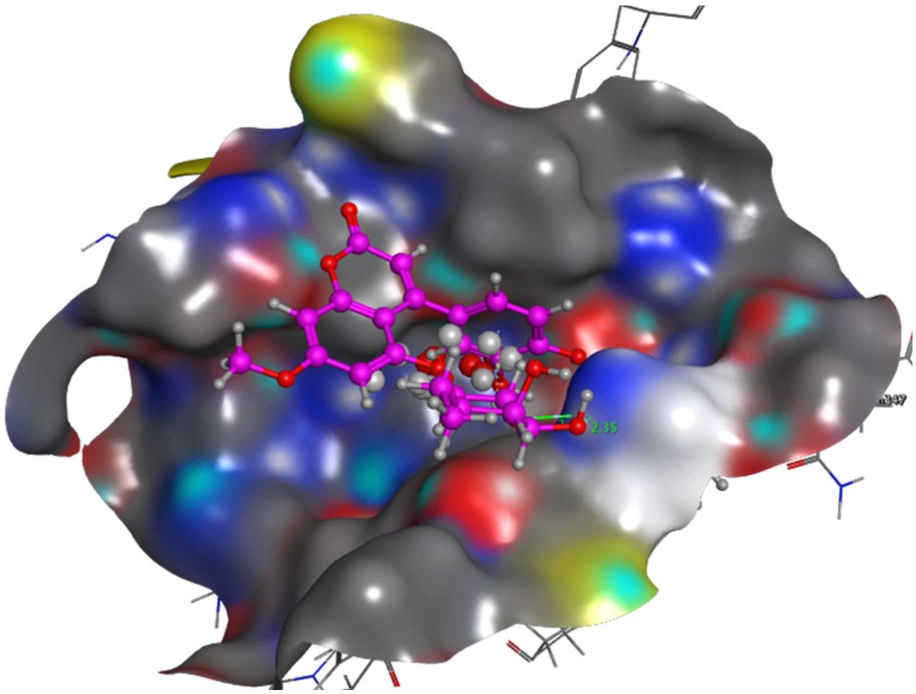

Figure S14 Ligand _LAIPNUDELSAV_31 in the binding site in endonucleases NSP-15

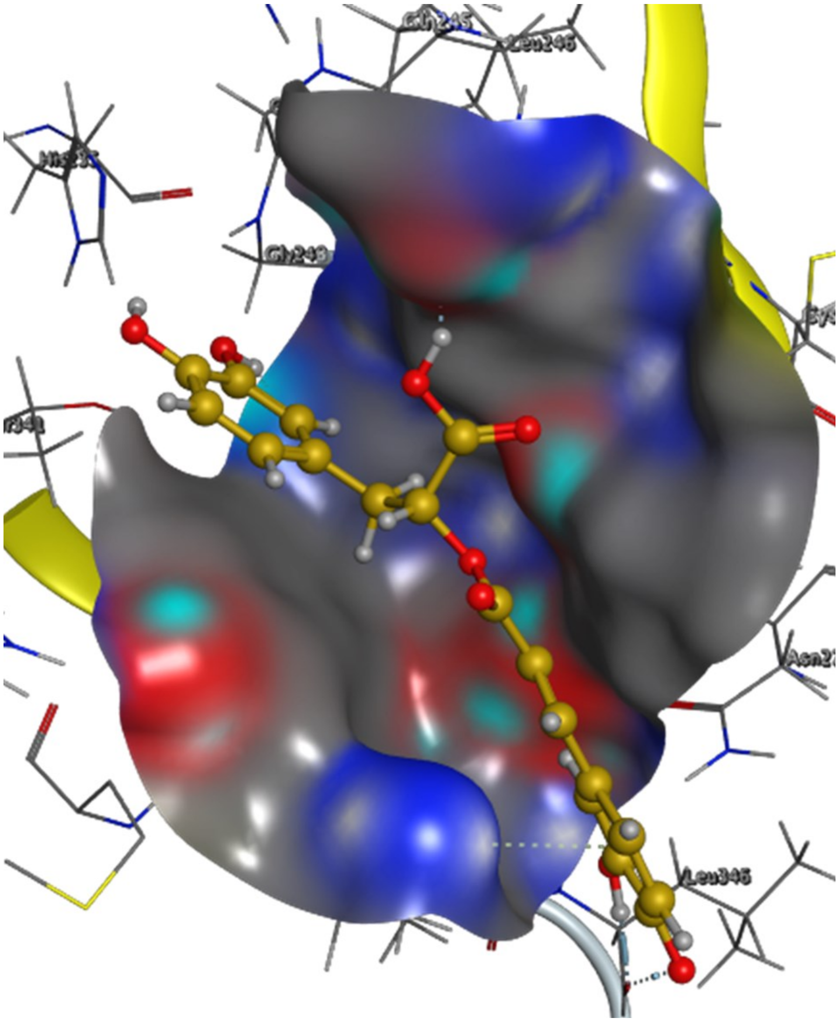

Figure S15 Ligand_UPMA_2V_266 in the site of protein NSP-15
